# Genomic survey of edible cockle (*Cerastoderma edule*) in the Northeast Atlantic: A baseline for sustainable management of its wild resources

**DOI:** 10.1111/eva.13340

**Published:** 2022-01-25

**Authors:** Manuel Vera, Francesco Maroso, Sophie B. Wilmes, Miguel Hermida, Andrés Blanco, Carlos Fernández, Emily Groves, Shelagh K. Malham, Carmen Bouza, The Cockle’s Consortium, Peter E. Robins, Paulino Martínez

**Affiliations:** ^1^ Department of Zoology, Genetics and Physical Anthropology ACUIGEN Group Faculty of Veterinary Universidade de Santiago de Compostela, Campus of Lugo Lugo Spain; ^2^ Institute of Aquaculture Universidade de Santiago de Compostela Santiago de Compostela Spain; ^3^ Department of Life Sciences and Biotechnologies University of Ferrara Ferrara Italy; ^4^ School of Ocean Sciences Marine Centre Wales Bangor University Menai Bridge UK

**Keywords:** 2b‐RAD, adaptive variation, fisheries management, genetic structure, larval dispersal modelling

## Abstract

Knowledge on correlations between environmental factors and genome divergence between populations of marine species is crucial for sustainable management of fisheries and wild populations. The edible cockle (*Cerastoderma edule*) is a marine bivalve distributed along the Northeast Atlantic coast of Europe and is an important resource from both commercial and ecological perspectives. We performed a population genomics screening using 2b‐RAD genotyping on 9309 SNPs localized in the cockle's genome on a sample of 536 specimens pertaining to 14 beds in the Northeast Atlantic Ocean to analyse the genetic structure with regard to environmental variables. Larval dispersal modelling considering species behaviour and interannual/interseasonal variation in ocean conditions was carried out as an essential background to which compare genetic information. Cockle populations in the Northeast Atlantic displayed low but significant geographical differentiation between populations (*F*_ST_ = 0.0240; *p* < 0.001), albeit not across generations. We identified 742 and 36 outlier SNPs related to divergent and balancing selection in all the geographical scenarios inspected, and sea temperature and salinity were the main environmental correlates suggested. Highly significant linkage disequilibrium was detected at specific genomic regions against the very low values observed across the whole genome. Two main genetic groups were identified, northwards and southwards of French Brittany. Larval dispersal modelling suggested a barrier for larval dispersal linked to the Ushant front that could explain these two genetic clusters. Further genetic subdivision was observed using outlier loci and considering larval advection. The northern group was divided into the Irish/Celtic Seas and the English Channel/North Sea, while the southern group was divided into three subgroups. This information represents the baseline for the management of cockles, designing conservation strategies, founding broodstock for depleted beds and producing suitable seed for aquaculture production.

## 1 INTRODUCTION

The genetic structure of marine species and patterns of connectivity between discrete populations is central to their health and resilience to external pressures such as parasites and pathogens (Rowley et al., 2014), pollution, exploitation and climate change over ecological and evolutionary timescales (Burgess et al., 2014; Cowen & Sponaugle, 2009). Detection of genetic structure in marine species remains challenging due to the often large effective population sizes and high levels of gene flow facilitated by the scarcity of physical barriers, which would be expected to lead to genomic homogenization (Danancher & Garcia‐Vazquez, 2011; do Prado et al., 2018; Zhao et al., 2018). However, genetic differentiation can be driven by features such as oceanographic barriers (e.g. current systems, fronts, gyres and eddies) or environmental gaps limiting dispersal (Blanco‐González et al., 2016; Nielsen et al., 2004; Vera et al., 2016; Xuereb et al., 2018), but also natural local selection in response to environmental variation (Clucas et al., 2019; Jiménez‐Mena et al., 2020; Vilas et al., 2015). Furthermore, historical vicariance, not yet erased by gene flow, and reproductive isolation can be other factors explaining differentiation (Gagnaire et al., 2015; Riginos et al., 2016; Saha et al., 2021). Distinguishing between neutral and adaptive genetic variation in the marine landscape has become a central issue in conservation biology, allowing for interpreting genetic variation in both historical/demographic and adaptive terms (Bernatchez, 2016; Nielsen et al., 2009). This information is essential for identifying the genetic diversity needed for conservation and breeding programmes in marine aquaculture (do Prado et al., 2018; Hughes et al., 2019).

The edible cockle, *Cerastoderma edule*, is a bivalve mollusc naturally distributed along the Northeast Atlantic coast, from Senegal to Norway, inhabiting intertidal soft sediments (Hayward & Ryland, 1995). The species plays a crucial role as a food source for birds, crustaceans and fish (Norris et al., 1998). Moreover, the species is highly appreciated for cuisine, and its main fisheries are located in Ireland, the United Kingdom, France, Spain and Portugal, where its commercialization drives employment of thousands of collectors, processors and sellers (http://www.cockles‐project.eu/). *Cerastoderma edule* is dioecious and can live up to 10 years in the wild with a fast sexual maturation (reached in its first year) and high fecundity (Honkoop & van der Meer, 1998), with its reproductive period spanning from late spring to mid‐autumn (Mahony et al., 2020; Malham et al., 2012). Larvae are planktonic and remain in the water column for around 30 days, which allows for larval dispersal by ocean currents that drive connectivity and gene flow between populations along the Northeast Atlantic coast (Dare et al., 2004; de Montaudouin et al., 2003).

The genetic structure of *C*. *edule* across the Northeast Atlantic has been studied over the last 40 years. Pioneering studies using allozymes detected genetic differences between populations located on either side of the English Channel (collected in Wales, France and The Netherlands; Beaumont et al., 1980), but also a high connectivity and gene flow from France to Denmark (Hummel et al., 1994), although other processes such as low genetic drift effect combined with recent population divergence may explain the observed patterns. Mitochondrial DNA (mtDNA) sequencing carried out on a wider sampling scale (from Morocco to Russia) revealed the presence of two major mtDNA groups in northern and southern areas, suggesting the presence of a northern cryptic refuge for *C*. *edule* (Krakau et al., 2012; Martínez et al., 2015). Studies using microsatellites showed high homogeneity in the southern beds from Portugal and Spain (Martínez et al., 2013, 2015), while two main clusters were identified in the northern area in the British Isles and the North Sea. In summary, three major areas were defined from microsatellite data: (i) a southern region (Morocco, Portugal, Spain and French beds up to the English Channel); (ii) Ireland, Great Britain and southern North Sea (the Netherlands and Germany); and (iii) a northern group (Scotland, Denmark, Norway and Russia) (Martínez et al., 2015). However, the low amount of microsatellite markers used has greatly limited the investigation of local adaptation and population connectivity at the fine scale necessary for the appropriate management of exploited species (Bernatchez et al., 2017).

Recently, Coscia et al. (2020) analysed the genetic structure and connectivity among cockle populations within the Celtic/Irish Seas using Restriction‐site Associated DNA sequencing (RADseq) data and a population genomics approach in combination with information on ocean conditions and larval dispersal modelling. They identified a significant genetic differentiation in the regional study area (three groups; *F*_ST_ = 0.021), mainly driven by salinity, larval dispersal by oceanographic currents and geographical distance. These results suggest that a finer structure can underlie cockle distributions in the Northeast Atlantic and that a genomic scan covering southern and northern beds combined with understanding of the dispersal of larvae in the area is necessary to understand how the species is structured for its appropriate management.

In this study, we applied a 2b‐RADseq genotyping by sequencing approach to assess the genetic structure of *C*. *edule* along the Northeast Atlantic coast considering environmental drivers and models of larval dispersal with the aim of providing essential information for the sustainable management of its resources. Major regions previously identified with microsatellites were confirmed, but refined information was obtained, mostly in agreement with the ocean dynamics and resulting larval dispersal patterns across the Northeast Atlantic.

## 2 MATERIALS AND METHODS

### 2.1 Oceanography of the study area

**FIGURE 1:**
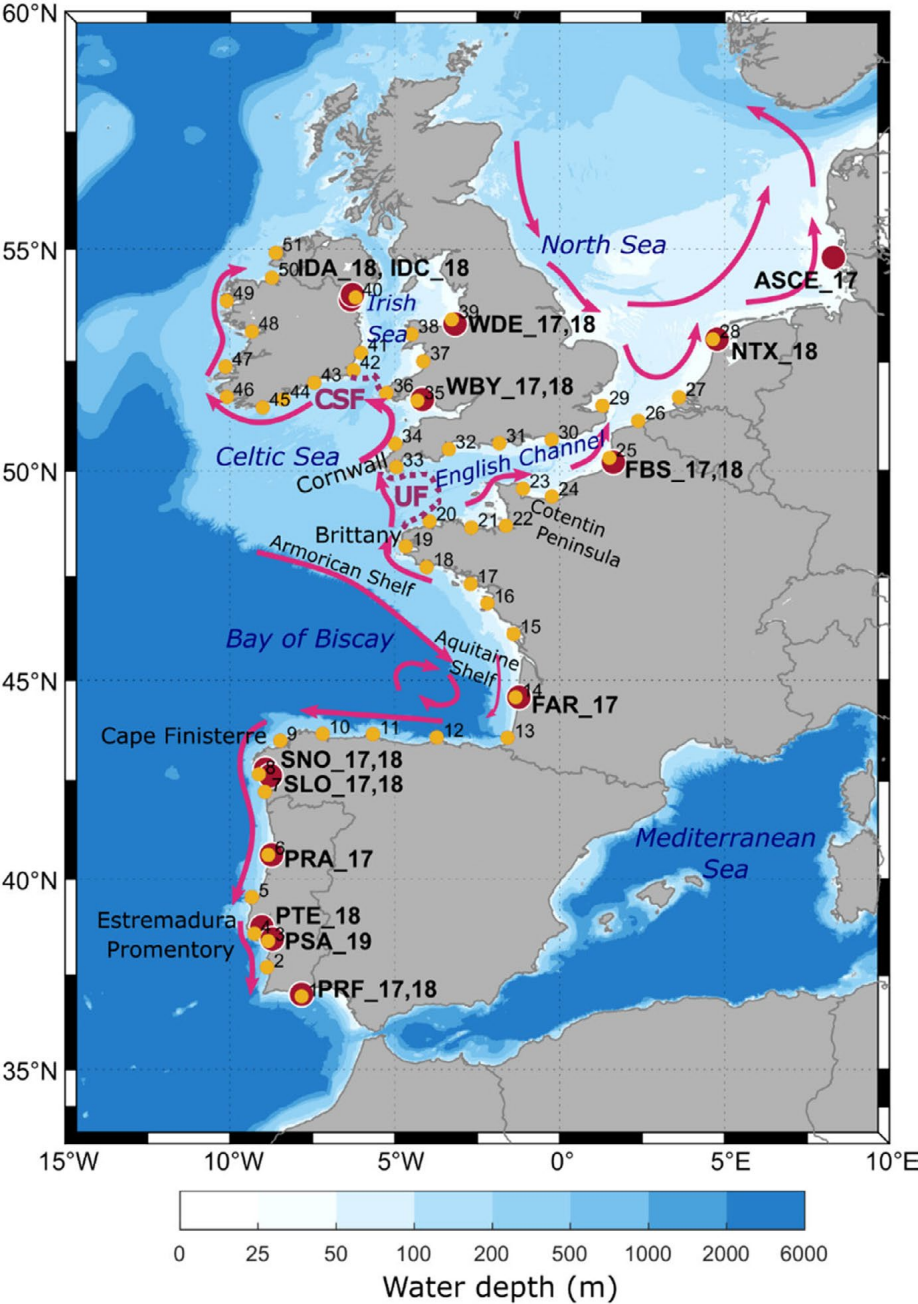
Study area for *Cerastoderma edule* genetic analysis and larval dispersal modelling. Ocean bathymetry is shaded in blue. Summer surface currents are schematically represented by magenta‐coloured arrows (see section Study Area for a detailed description and references). Locations of fronts are depicted by purple dotted lines (CSF, Celtic Sea Front; UF, Ushant Front). Location of the *C. edule* beds for the genetic analysis are shown in dark red (see Table 1 for location codes) and particle release locations for larval dispersal modelling are shown in yellow and numbered from 1 to 51. Location codes in panel A are detailed in Table 1

The study area covers the Northeast Atlantic from southeast Portugal to northeast Ireland and the southern North Sea (Figure 1). This area is divided into several oceanographic regions: Iberian coastal waters, the Bay of Biscay, the English Channel, the Celtic Sea, the Irish Sea and the North Sea. These regions are to some extent discrete units with limited oceanographic connectivity between them resulting from either divergent coastal currents or frontal currents generated during summer heating (Galparsoro et al., 2014). During summer months (corresponding to the spawning period of *C*. *edule*), when upwelling is a prominent feature along the Galician coast (NW Spain), the Portuguese coastal currents transport surface waters southward along the west coast of the Iberian Peninsula, whereas during the winter months the Iberian Poleward Current shoals and moves surface waters northwards (Teles‐Machado et al., 2016). Along the southern Bay of Biscay, a strong westward transport develops during the summer months which changes direction during the winter months and links into the slope current along the Armorican and Aquitaine Shelf (W France). The coastal circulation along the east Bay of Biscay is characterized by northward transport by the Iberian Poleward Current during the winter, which reverses in direction and reduces in strength during the summer months (Charria et al., 2013). Tidal mixing fronts separating mixed from seasonally stratified waters form in early summer and extend into the autumn at the eastern entrance to the English Channel (Ushant Front) Group “Grepma” (1988) and between the Celtic and the Irish Sea (Celtic Sea Front). From late spring to early autumn, a current system develops in the Celtic Sea that transports waters from southwestern Britain via the frontal jet associated with the Celtic Sea Front to the south and west coasts of Ireland as the Irish coastal current (Brown et al., 2003; Fernand et al., 2006; Horsburgh et al., 1998). Flow through the English Channel is generally north‐eastward and links into the anticyclonic circulation of the North Sea, which is characterized by a southward flow along the east‐coast of the UK that joins into the coastal current transporting waters northward along the southern North Sea coastlines and the Norwegian Coastal Current (Winther & Johannessen, 2006).

### 2.2 Sampling

A total of 545 individuals from 14 cockle beds distributed along the Northeast Atlantic Coast (Figure 1) were collected during the period 2017–2019 and stored in 100% ethanol (Table 1). Temporal replicates, to analyse genetic stability across generations, were obtained for six beds in 2017 and 2018 (Table 1). All specimens belonged to the 0+ class of the year, and they were collected to avoid generation overlapping between consecutive year cohorts.

**TABLE 1:** Sample details and genetic diversity features of *Cerastoderma edule* beds studied in the Northeast Atlantic region

Bed	Sampling year	Latitude (°N)	Longitude (°E)	Code	Country	*N*	Complete data set			Polymorphic loci			
							*H*_o_	*H*_e_	*F*_IS_	No. of polymorphic loci (% total)	*H*_o_	*H*_e_	*F*_IS_
Sylt	2017	54.814	8.298	ASCE_17	Germany	22 (22)	0.070	0.077	0.089	2402 (25.8)	0.172	0.191	0.099
Texel	2018	53.004	4.771	NTX_18	Netherlands	21 (21)	0.075	0.078	0.034	2662 (28.6)	0.183	0.193	0.052
Dee Estuary	2017	53.343	−3.174	WDE_17	Wales	30 (28)	0.071	0.078	0.079	2625 (28.2)	0.165	0.182	0.093
Dee Estuary	2018	53.343	−3.174	WDE_18	Wales	30 (30)	0.075	0.083	0.099	3891 (41.8)	0.153	0.172	0.110
Burry	2017	51.643	−4.166	WBY_17	Wales	30 (30)	0.073	0.081	0.092	3596 (38.6)	0.148	0.165	0.103
Burry	2018	51.643	−4.166	WBY_18	Wales	30 (30)	0.070	0.077	0.098	2509 (27.0)	0.157	0.176	0.108
Dundalk Bay‐Annagassan	2018	53.884	−6.341	IDA_18	Ireland	29 (29)	0.074	0.081	0.083	3027 (32.5)	0.159	0.176	0.097
Dundalk Bay‐Cooley	2018	53.996	−6.287	IDC_18	Ireland	22 (22)	0.077	0.084	0.084	3569 (38.3)	0.173	0.190	0.089
Somme Bay	2017	50.201	1.627	FBS_17	France	30 (30)	0.072	0.080	0.110	3479 (37.4)	0.146	0.166	0.120
Somme Bay	2018	50.201	1.627	FBS_18	France	31 (31)	0.072	0.079	0.084	3139 (33.7)	0.149	0.165	0.097
Archachon Bay	2017	44.580	−1.238	FAR_17	France	30 (30)	0.074	0.083	0.111	4420 (47.5)	0.138	0.157	0.121
Ría de Noia	2017	42.790	−8.923	SNO_17	Spain	30 (30)	0.079	0.088	0.098	4970 (53.4)	0.139	0.155	0.103
Ría de Noia	2018	42.790	−8.923	SNO_18	Spain	30(30)	0.072	0.081	0.118	3716 (39.9)	0.137	0.158	0.133
Lombos do Ulla	2017	42.629	−8.775	SLO_17	Spain	30 (30)	0.076	0.086	0.114	4700 (50.5)	0.136	0.155	0.123
Lombos do Ulla	2018	42.629	−8.775	SLO_18	Spain	32 (32)	0.073	0.083	0.119	4237 (45.5)	0.134	0.153	0.124
Ria de Aveiro	2017	40.623	−8.739	PRA_17	Portugal	30 (30)	0.072	0.081	0.113	3516 (37.8)	0.141	0.161	0.124
Tejo Estuary	2018	38.767	−9.033	PTE_18	Portugal	8 (8)	0.073	0.078	0.063	1886 (20.3)	0.271	0.290	0.066
Sado Estuary	2019	38.450	−8.717	PSA_19	Portugal	20 (20)	0.071	0.080	0.108	2378 (25.5)	0.177	0.202	0.124
Ria Formosa	2017	36.998	−7.830	PRF_17	Portugal	30 (30)	0.079	0.084	0.066	4250 (45.7)	0.146	0.158	0.076
Ria Formosa	2018	36.998	−7.830	PRF_18	Portugal	30 (23)	0.079	0.086	0.079	4145 (44.5)	0.159	0.174	0.086

Abbreviations: *F*_IS_, intrapopulation fixation index; *H*_e_, expected heterozygosity; *H*_o_, observed heterozygosity; *N*, number of individuals analysed (in parentheses *N* after quality filtering).

### 2.3 Single nucleotide polymorphism genotyping

Total DNA was extracted from gill tissue samples using the e.Z.N.A. E‐96 mollusc DNA kit (OMEGA Bio‐tech), following manufacturer recommendations. Single nucleotide polymorphism (SNP) identification and selection, as well as genotyping and validation protocols followed those described by Maroso et al. (2019). Briefly, AlfI IIb restriction enzyme (RE) was used to construct the 2b‐RAD libraries, which were evenly pooled for sequencing in Illumina Next‐seq including 90 individuals per run. The recently assembled cockle's genome (794 Mb; A. Bruzos, J. M. C. Tubio, unpublished data) was used to align reads from each individual using Bowtie 1.1.2 (Langmead et al., 2009), allowing a maximum of three mismatches and a unique valid alignment (‐v 3 ‐m 1). Individuals with <250,000 reads were discarded. STACKS 2.0 (Catchen et al., 2013) was then used to call SNPs and genotype a common set of markers in the sample set, applying the marukilow model with default parameters in the gstacks module of Stacks 2.0. This SNP panel was further filtered by applying the following criteria: (i) genotyped in >60% individuals in the total sample; (ii) minimum allele count (MAC) ≥3 in the total sample; (iii) conformance to Hardy–Weinberg equilibrium within each sample (HWE) across the whole collection; that is, loci with significant deviation from HWE (*p* < 0.05) in more than 25% of samples were removed; and (iv) selection of the most polymorphic SNP in each RAD‐tag.

### 2.4 Genetic diversity and population structure

Genetic diversity (i.e. observed (*H*_o_) and expected (*H*_e_) heterozygosities, proportion of polymorphic loci), departure from Hardy–Weinberg equilibrium (HWE) and intrapopulation fixation index (*F*_IS_) were estimated for each bed using GENEPOP v4.0 (Rousset, 2008) and ARLEQUIN v3.5 (Excoffier & Lischer, 2010). *H*_e_, *H*_o_ and *F*_IS_ were also calculated for each population using only markers that were polymorphic within each bed (MAC ≥ 2). Linkage disequilibrium between pairs of loci regarding physical distance (bp) was estimated with *r* ^2^ across the cockle genome using PLINK 1.9 (Chang et al., 2015; http://www.cog‐genomics.org/plink/1.9), and its significance calculated through exact tests over genotypic contingency tables using GENEPOP v4.0.

Global and pairwise relative coefficients of population differentiation (*F*_ST_) between cockle beds were calculated with ARLEQUIN v3.5 using 10,000 permutations to test for significance. The number of genetically homogenous population units (*K*) was estimated using the variational Bayesian clustering method implemented in the package fastSTRUCTURE v2.3.4 (Raj et al., 2014) for *K* = 1 − number of samples + 1, with an admixture ancestry model, convergence criterion of 1 × 10^−7^, five cross‐validated sets and the simple prior (flat‐beta prior). The most likely number of *K* was estimated using the ‘chooseK.py’ programme. This programme gives the best *K* value and *K* corresponding with weak population structure in the data using the heuristic scores. Summarized outputs were carried out using the software DISTRUCT 1.1 (Rosenberg, 2004). Discriminant analyses of principal components (DAPC) were run in ADEGENET package (Jombart et al., 2010; Jombart & Ahmed, 2011) for the R platform (R Development Core Team, 2014; http://www.r‐project.org/). Data were transformed using PCA (principal component analysis), and an appropriate number of principal components (PC) and discriminant functions (DF) were retained to explain >90% of the variance. Analyses of molecular variance (AMOVA) to study the distribution of genetic diversity within (*F*_SC_) and among (*F*_CT_) bed groups obtained from fastSTRUCTURE analyses were carried out with the program ARLEQUIN v.3.5 and their significance tested with 10,000 permutations. Isolation by distance (IBD) was evaluated by the correlation between geographical (measured as the shortest ocean distance) and genetic (measured as *F*_ST_/1 − *F*_ST_; Rousset, 1997) distance matrices checked with a Mantel test with 10,000 permutations using NTSYS v.2.1 (Rohlf, 1993). All these analyses were performed with the complete SNP data set (9309 markers), the neutral data set (8021 markers) and the detected divergent outlier loci data set (554 markers) (see Section 3). Finally, the search algorithm implemented in the TREEMIX program (Pickrell & Pritchard, 2012) was used to infer population history of cockle beds. The program creates a maximum‐likelihood phylogenetic tree that incorporates admixture on the basis of allele frequencies and a Gaussian approximation to genetic drift, allowing patterns of splits and mixtures in multiple populations to be inferred. The different beds were used as independent clusters, grouping the temporal replicates, and only the neutral data set was used. To avoid biases linked to missing data, the neutral data set was further filtered for this analysis to retain only markers shared by at least 90% of the individuals (2940 SNPs). To account for linkage disequilibria, SNPs were grouped in windows of five markers. Given the density of markers used, this number ensured that markers in different windows were unlinked (see below). A tree without migration events was initially generated; then, migration events were sequentially added one by one up to 14, and the likelihood was monitored to check when it reached a plateau for estimating the number of migration events.

### 2.5 Outlier tests and gene mining

Several statistical approaches were applied to identify outlier loci subject to selection in different geographic scenarios, namely the whole Northeast Atlantic region and the two main northern and southern groups identified by fastSTRUCTURE (see Section 3). The Bayesian *F*_ST_‐based method implemented in BAYESCAN v2.01 (Foll & Gaggiotti, 2008) was used by grouping individuals by beds in all the analyses performed. BAYESCAN was run using default parameters (i.e. 20 pilot runs; prior odds value of 10; 100,000 iterations; burn‐in of 50,000 iterations and a sample size of 5000). Additionally, the FDIST *F*_ST_ method implemented in ARLEQUIN v3.5, which uses a maximum‐likelihood approach (Beaumont & Nichols, 1996), was applied to incorporate a priori information regarding population structure. Thus, two different scenarios were tested for the whole Atlantic region using this methodology: (i) grouping individuals by beds (locations: LOC scenario, as in BAYESCAN analysis); and (ii) grouping beds by groups identified by STRUCTURE using a hierarchical island model (HIER scenario). All ARLEQUIN runs were carried out with 50,000 simulations, 10 groups and 100 demes per group. All these strategies can be affected by type I (false positive) or type II (false negative) errors. The methodology used by BAYESCAN is more conservative than ARLEQUIN one (Narum & Hess, 2011). For this reason, loci with a false discovery rate (FDR, *q*‐value) <0.05 were considered consistent outliers with this methodology. For ARLEQUIN analysis, loci at *p* < 0.01 were considered consistent outliers, while loci at *p* < 0.05 were just only suggestive (Vera et al., 2019). Accordingly, all loci without any signal of being under selection (neither suggestive or consistent) were considered as neutral, while for the outlier analysis in the different geographical scenarios, only the consistent outliers were considered, namely those at *p* < 0.05 and *p* < 0.01 with BAYESCAN and ARLEQUIN respectively.

RAD‐tags including divergent outlier SNPs were mapped in the *C*. *edule* assembled genome (A. Bruzos, J. M. C. Tubio, unpublished data; Scuba Cancers ERC‐2016‐STG project) and used as landmarks for mining the genome to identify candidate genes related to selective drivers. The total genome size (794 Mb) was mostly assembled (95.2%) in the 19 mega‐scaffolds (chromosomes: C onwards) corresponding to the cockle's haploid karyotype (Insua & Thiriot‐Quiévreux, 1992). To establish the size of the windows for genome mining and the reliability of each region under selection, linkage disequilibrium (LD) was evaluated across the cockle's genome within each cockle bed. Furthermore, we also evaluated LD using the whole data set, keeping in mind population structure at genomic islands of differentiation produces enhanced LD. It was expected that those regions under selection would show a higher LD than the average due to selective sweeping. LD was represented against physical distance for all pairs of markers within each chromosome across the whole genome using PLINK 1.9, between markers separated up to 5000 kb in each bed and up to 500 kb in the whole cockle collection of the Northeast Atlantic respectively. The corresponding *r* ^2^ values (the square of the correlation coefficient checking for the nonrandom association of alleles at pairs of loci) were averaged within 50 and 1 kb genomic windows within each bed and for the whole sampling collection respectively. Considering that LD was negligible above 50 kb in the whole collection (see Section 3), we established windows of ±250 kb around the outlier markers in the selected genomic regions for mining following a conservative criterion and taking into account that selective sweeps could increase LD in those genomic regions. The most consistent regions under selection were identified according to the following criteria: (i) the presence of two or more consecutive outliers (seed) across the whole SNP data set mapped in the cockle genome; the most external outliers of the seed were taken as reference to expand the window ±250 kb; and if a new outlier lay in that region, then the window was further expanded other 250 kb; (ii) significant *p* values of pairwise genotypic disequilibrium between the outlier loci in the window; and (iii) magnitude of the LD itself (average *r* ^2^ between outlier pairs in the window). Genes included in those genomic windows were identified using the cockle's transcriptome assembled and annotated using RNAseq data from a study related to resistance to *Marteilia cochillia* within the COCKLES EAPA_458/2016 project (B. G. Pardo, C. Fernández, P. Martínez, unpublished data) and the cockle's genome (A. Bruzos, J. M. C. Tubio, unpublished data). The annotated cockle's transcriptome was used as reference to detect Gene Ontology (GO) functional enrichment of the genomic regions under selection (FDR 5%) using agriGO v2.0 (Tian et al., 2017). A certain bias could be expected since the digestive gland, the target organ of *M*. *cochillia*, was used for RNAseq in most individuals, so results should be taken with caution.

### 2.6 Landscape analyses

Genetic differentiation explained by the different spatial (latitude and longitude) and abiotic factors (see below) was studied following a canonical redundancy analysis (RDA) using the VEGAN software (Oksanen, 2015) in R. For each bed, allele frequencies were estimated with ADEGENET package in R (Jombart & Ahmed, 2011) using the ‘makefreq’ option applied on the ADEGENET ‘genpop’ file. Only information for the year 2017 was used, so temporal samples from the 2018 cohort were not considered. Loci with missing values were excluded from the analysis. Latitude and longitude together with the following abiotic factors were available for all the beds except for ASCE_17 (Sylt‐Germany): sea surface temperature (SST, °C); sea bottom temperature (SBT, °C); sea surface salinity (SSS, psu); sea bottom salinity (SBS, psu); bottom shear stress (BSS, N m^−2^); and net primary productivity (NPP, mg m^−3^ day^−1^) (Table S1). Monthly mean information for all these abiotic factors was retrieved from the IBI_REANALYSIS_PHYS_005_002 ocean reanalysis model (https://resources.marine.copernicus.eu/?option=com_csw&task=results?option=com_csw&view=details&product_id=IBI_REANALYSIS_PHYS_005_002") and the IBI_REANALYSIS_BIO_005_003 model (https://resources.marine.copernicus.eu/?option=com_csw&task=results?option=com_csw&view=details&product_id=IBI_REANALYSIS_BIO_005_003) for the period 2014–2018. The nearest model cell with water was taken to extract the data. Then, averages for the two seasonal extremes, winter (i.e. from January to March) and summer (i.e. from July to September), and for the spawning season (i.e. from April to September, see Malham et al., 2012; Mahony et al., 2020) were calculated for each bed.

ANOVA was performed to test the significance of the variance associated with the different variables using 1000 random permutations with VEGAN. The variance inflation factor (VIF) was estimated to explore collinearity (correlation) among landscape variables in the data set. VIF values higher than 10 would denote important collinearity problems, and values from 5 to 10 moderate problems, while values lower than 5 would indicate no collinearity problems (Marquardt, 1970). Different models were adjusted following a forward selection process with the PACKFOR package in R (Dray et al., 2009). This selection process corrects for highly inflated type I errors and overestimated amounts of explained variation (Vandamme et al., 2014). Thus, the reduced panel of explanatory variables is used to recalculate the total proportion of genetic variation in the variance partitioning. The weight of the different loci on the significant environmental vectors was obtained using VEGAN. All these analyses were performed separately for the complete, neutral and divergent outlier SNP data sets for the whole Northeast Atlantic region.

### 2.7 Larval dispersal modelling

A larval dispersal model was developed for the Northeast Atlantic, in which virtual particles representing cockle larvae were ‘released’ from cockle bed locations along the Atlantic coast and transported by simulated ocean currents for the duration of their assigned pelagic larval phase. The particle trajectories were tracked enabling us to estimate the likely larval dispersal patterns and potential connectivity between different cockle populations.

Simulated ocean velocities were extracted from the IBI (Iberian Biscay Ireland) Ocean Analysis and Forecast system (IBI_ANALYSIS_FORECAST_PHYS_005_001; see https://resources.marine.copernicus.eu/?option=com_csw&view=details&product_id=IBI_ANALYSIS_FORECAST_PHYS_005_001 for details and to download the data). The underlying hydrodynamic model is based on the NEMO ocean model, version 3.6. The primitive equations are solved on a horizontal grid with a resolution of 1/36° and 50 unevenly spaced vertical levels with resolution decreasing from the surface into the deep. GEBCO08 was used for the bathymetry. Atmospheric forcing was extracted from ERA interim atmospheric fields. At the lateral open boundaries, ocean forcing fields were obtained from the global CMEMS GLOBAL eddy resolving system at 1/12°, and tidal forcing from the global FES2014 database (https://www.aviso.altimetry.fr/en/data/products/auxiliary‐products/global‐tide‐fes.html). Freshwater fluxes from rivers were implemented for 33 rivers across the model domain. The model has been extensively validated and quality controlled (Sotillo et al., 2015, 2020).

For the larval dispersal model, particles were released from 51 coastal sites (see Figure 1 for numbered release sites) covering 11 of the 14 wild natural beds sampled (11 because two sets of sample sites were in close proximity to one another, and one site was not within the ocean model domain), plus 40 other sites where cockles are known to habit (Chust et al., 2013; see figures 3 and 4 in Mahony et al., 2020; Rivadulla et al., 2017). These 51 sites were roughly evenly distributed along the Atlantic coastline (ca. 60–150 km apart), hence reducing bias in the connectivity modelling from large differences in spatial distances between sites. Tests were carried out adding and removing sites in order to prevent creating artificial breaks in dispersal (not shown).

From each site, cohorts of 400 particles were released for the first 16 days of each month from April to September, corresponding to the spawning phase of *C*. *edule* (Mahony et al., 2020; Malham et al., 2012), for the years 2016 to 2018 in order to capture tidal phase variations, intra‐annual and interannual variability due to changes in the formations, positions and strengths of the coastal and frontal currents. Larvae were released at three different depth levels (1, 15 and 30 m water depth) to account for the large uncertainties of larval behaviour (see below for details) of *C*. *edule* and advected for 40 days at each level. The last 10 days of the simulation were used for analysis, thus accounting for uncertainties in the length of the pelagic larval phase.

While it is possible that spawning varies latitudinally, a recent literature and data review (Mahony et al., 2020) found no clear evidence of such a pattern. We therefore apply the same spawning period for all sites. At present, very little is known about cockle larval behaviour. Existing studies show that bivalve larvae appear not to be randomly dispersed throughout the water column but are aggregated at certain depths in the water column (mostly between 10 and 40 m) (Irigoien et al., 2004; Mann, 1985; Raby et al., 1994; Scrope‐Howe & Jones, 1986; Tremblay & Sinclair, 1990) with a number of studies showing an association of bivalve larvae with the thermocline (Raby et al., 1994; Scrope‐Howe & Jones, 1986; Tremblay & Sinclair, 1990). The approach taken here allowed us to explore different scenarios of dispersal focussing on the ocean current dispersal at different depths rather than on very uncertain and potentially even ‘artificially’ induced larval behaviours that are not backed by evidence from real ocean scenarios.

Results are presented in terms of probability distribution maps and connectivity networks. To calculate connectivities, it was assumed that the larvae were able to settle during the last 10 days of their larval stage (i.e. days 31–40); therefore, each particle's positions during this time were used for the analysis of larval connectivities between sites. Larval connectivities were calculated as the percentage of larvae released from the source location arriving within a square of 0.2° latitude/longitude distance of one of the other 50 sink locations or returning to the source location (self‐recruitment). Connectivities were averaged over the three vertical release levels, but also presented separately in the Supporting Information.

## 3 RESULTS

### 3.1 SNP genotyping and genetic diversity within beds

A total of ~2000 M raw reads were sequenced (~3.7 M reads per sample). After quality filtering and alignment to the cockle genome, 27.7% and 35.6% total reads, respectively, were removed, and ~750 M reads were fed into STACKS to yield 315,744 loci with 726,911 SNP positions. Filtering was mostly due to multiple matches in the cockle's genome. Nine individuals (two from Dee Estuary—WDE_17—and seven from Ria Formosa—PRF_18; Table 1) with low number of reads (<250,000 reads) were removed. Thus, a total of 536 individuals from 14 beds were retained and used for subsequent analyses. After filtering by population criteria, the number of retained SNPs for the whole population sample was 9309. Most of these SNPs mapped on the 19 cockle chromosomes (97.5%) corresponding to the haploid chromosome number of the species (Insua & Thiriot‐Quiévreux, 1992), the remaining mapping on small scaffolds (A. Bruzos, J. M. C. Tubio, unpublished data) (Table S2).

Genetic diversity per sample was estimated using the whole SNP data set as the average observed (*H*_o_) and expected heterozygosity per locus (*H*_e_). *H*_o_ ranged from 0.070 in Sylt—ASCE_17 and Burry—WBY_18 to 0.079 in Ría de Noia—SNO_17, Ría Formosa—PRF_17 and PRF_18 (mean ± SD = 0.074 ± 0.003), while *H*_e_ varied from 0.077 in Sylt—ASCE_17 and Burry—WBY_18 to 0.088 in SNO_17 (mean = 0.081 ± 0.003) (Table 1). *F*_IS_, which estimates the deviation from Hardy–Weinberg proportions within populations, was positive but not significant in all samples (i.e. heterozygote deficit; <0.119; Table 1), so random mating sustains in cockle beds from the Northeast Atlantic. The number of polymorphic loci, considered as those loci with MAC ≥2, ranged from 1886 (20.2% of the total number of SNPs) in Tejo Estuary—PTE_18 to 4970 (53.4%) in Ría de Noia—SNO_17 (mean ± SD = 3455.9 ± 858.9). Genetic diversity increased when only polymorphic loci were taken into account: *H*_o_ varied from 0.134 in Lombos do Ulla—SLO_18 to 0.271 in Tejo Estuary—PTE_18 (mean ± SD = 0.159 ± 0.030) and *H*_e_ ranged from 0.153 in Lombos do Ulla—SLO_18 to 0.290 in Tejo Estuary—PTE_18 (mean ± SD = 0.177 ± 0.030). Similar to the *F*_IS_ estimations considering all SNPs, *F*_IS_ values using SNPs with MAF >0.01 were positive and nonsignificant in all cases (<0.124; Table 1).

### 3.2 Outlier detection and gene mining

Detection of SNPs under selection was addressed using a number of statistical approaches in different geographical scenarios to test whether the genetic differentiation between populations for each SNP was above or below the neutral background (outlier loci), and thus suggestive of divergent or balancing selection, respectively. In the whole Northeast Atlantic region, BAYESCAN detected a total of 460 outlier loci potentially under selection, 19 under balancing selection (BS) and 441 under divergent selection (DS) (FDR <0.05). The two ARLEQUIN scenarios analysed in the same region (i.e. LOC and HIER) detected 308 (293 DS and15 BS) and 278 (274 DS and 4 BS) consistent outliers (*p* < 0.01). These figures greatly increased when suggestive outliers (i.e. *p* < 0.05) were considered, which suggests a considerable proportion of false positives with this approach as previously suggested (Narum & Hess, 2011) (Table 2). BAYESCAN analyses within northern and southern groups identified by fastSTRUCTURE (see next section) did not detect outliers under balancing selection, while ARLEQUIN detected only six in the northern group (*p* < 0.01). The total number of consistent balancing outliers across the different approaches was 36 (Figure S1). Considering all approaches, the number of consistent divergent outliers detected for the Northeast Atlantic region, the northern group and the southern group were 554, 213 and 263, respectively, representing a total of 746 (Figure S1). A considerable number of outlier loci in the whole region overlapped with those detected in northern and southern groups. Nearly twice as many outlier loci were unique to the southern region (129) as to the northern one (61), which suggested more specific environmental drivers in the southern group. On the other hand, a very small number of outliers related to balancing selection were detected, most of them being specific of the different statistical approaches or geographical scenarios. Finally, after discounting all SNPs with any sign of being under selection, the neutral data sets were composed of 8021, 8570 and 8650 markers for the Northeast Atlantic region, the northern group and the southern group, respectively. These sets of markers, together with the whole SNP data set (9309 SNPs), were used for the subsequent analyses on population structure.

**TABLE 2:** Outliers identified by different statistical methodologies in *Cerastoderma edule* from Northeast Atlantic region

	BAYESCAN		ARLEQUIN				Total Div outliers	Total outliers
			Locations (beds)		Hierarchical (Northern vs. Southern groups)			
	Balancing	Divergent	Balancing	Divergent	Balancing	Divergent		
North‐eastern Atlantic	19	441	15 (426)	293 (599)	4 (282)	274 (705)	554	1288
Northern group	0	109	6 (181)	207 (557)	—	—	213	739
Southern group	0	64	0 (38)	259 (621)	—	—	263	659

Note

Number of outliers detected with BAYESCAN (FDR < 0.05) and ARLEQUIN (*p* < 0.01; *p* < 0.05 in parentheses) under balancing and divergent selection; Div outliers: total consistent divergent outlier identified by BAYESCAN and/or ARLEQUIN; Total outliers: all outliers detected with any selection signal in all the geographical scenarios tested.

**FIGURE 2:**
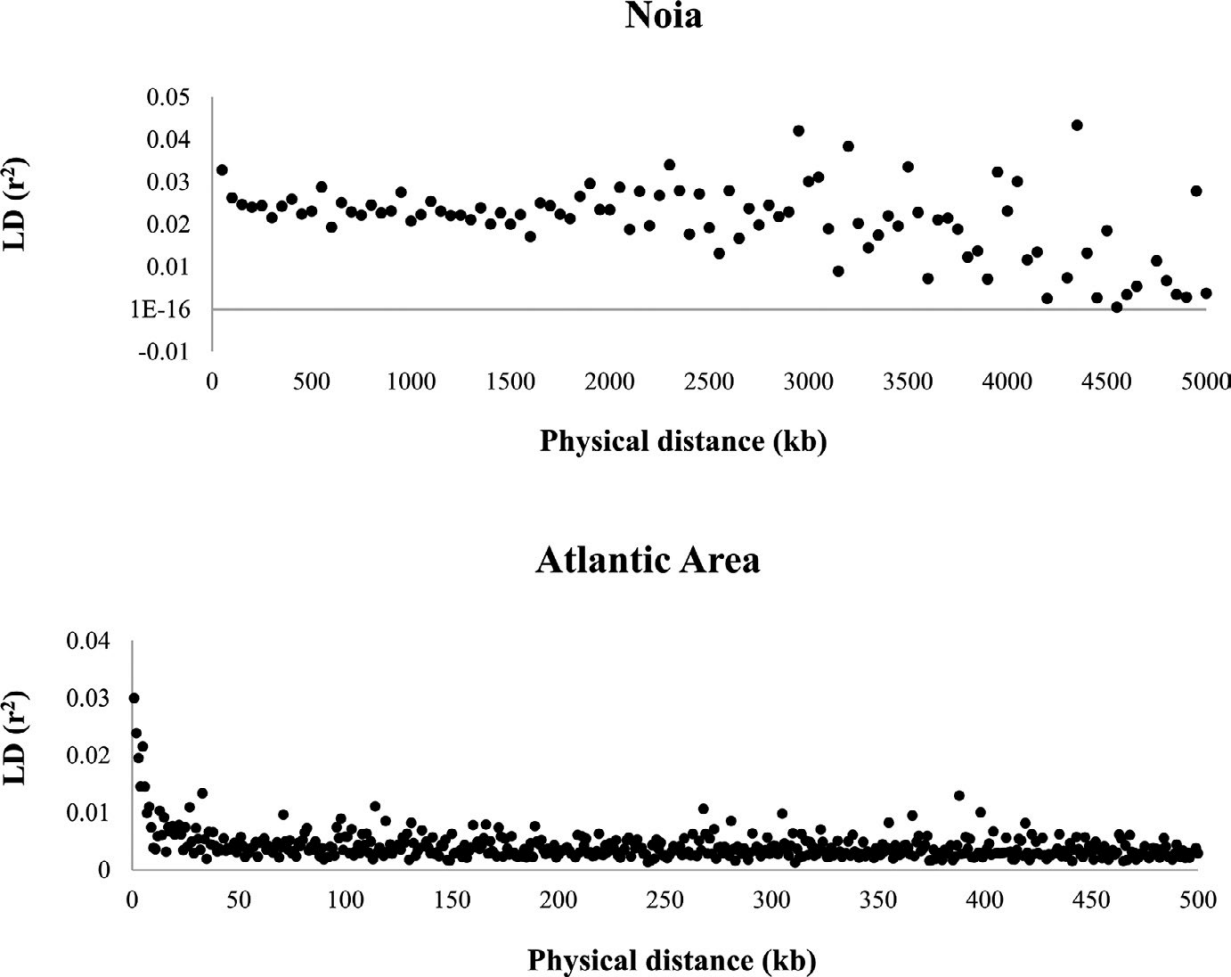
Linkage disequilibrium (*r* ^2^) with regard to physical distance between pairs of markers in the *Cerastoderma edule* genome in a representative cockle bed (Noia) and using the whole data set from the Atlantic Area

All the consistent outliers (36 BS and 746 DS) were mapped on the *C*. *edule* genome. Divergent outliers were distributed across the 19 chromosomes of its assembly (Table 3). Only 16 outliers mapped on minor scaffolds of the cockle's assembly and were not further used for mining considering its doubtful position. The proportion of outlier loci over those mapping within each chromosome was rather evenly distributed across chromosomes, between 6.2% in C14 and 10.6% in C13, excluding C18 where only 2.4% outliers were detected. The cockle's genome was used to estimate LD between adjacent markers regarding physical distance across the cockle's genome within each population as well as in the whole cockle collection of the Northeast Atlantic (Figure 2). Results showed that LD was on average very low both within each population as well as in the whole studied area and the highest average LD per window measured as *r* ^2^ was always below 0.050, even for very short distances. Within each population, LD was not significant for nearly all pairwise comparisons, in part due to the limited sample size (*N* ~ 30), and even when all data from the Northeast Atlantic was pooled (580 individuals), LD was mostly negligible and not significant above 50 kb on average (mean *r* ^2^ for all loci pairwise comparisons within chromosomes: 0.0045 ± 0.0001). Despite the low LD detected on average on the whole genome, we decided to be cautious in establishing the size of the windows for mining (±250 kb), considering that specific regions under selection should display significant LD across wider regions due to sweeping. Using the whole population data, 56 genomic windows under divergent selection characterized by the presence of two or more consecutive outliers were identified, encompassing a total of 31.0 Mb (3.9% of the whole genome) (Table 3; Tables S3 and S4). A total of 446 genes were identified in those windows, which showed significant enrichment (FDR 5%) for GO terms related to different metabolic processes such as heterocycle catabolic process (BP: GO:0046700), endopeptidase inhibitor activity (MF: GO:0004866), peptidase inhibitor activity (MF: GO:0030414) and enzyme inhibitor activity (MF: GO:0004857).

**TABLE 3:** Outlier loci and associated genomic regions under divergent selection across the *Cerastoderma edule* chromosomes according to the different scenarios tested in the North East Atlantic region

Chromosome	Chromosome size (Mb)	No. of mark	No. of outliers (%)	No. of consistent outliers (%)	Sum of genomic windows (bp)	No. of windows	No. genes in windows	No. of consistent outliers (%)					
								T	N	S	T&N	T&S	N&S&T (N&S)
1	64,609,245	785	76 (9.7)	23 (30.3)	2,726,662	4	35	11 (47.8)	0 (0.0)	2 (8.7)	6 (26.1)	3 (13.0)	1 (4.3)
2	56,319,168	729	54 (7.4)	6 (11.1)	886,053	2	14	4 (66.7)	0 (0.0)	1 (16.7)	1 (16.7)	0 (0.0)	0 (0.0)
3	55,987,847	749	60 (8.0)	23 (38.3)	3,181,048	5	58	10 (43.5)	0 (0.0)	3 (13.0)	5 (21.7)	5 (21.7)	0 (0.0)
4	52,087,795	544	50 (9.2)	12 (24.0)	1,543,748	1	20	4 (33.3)	0 (0.0)	1 (8.3)	6 (50.0)	1 (8.3)	0 (0.0)
5	50,828,891	703	53 (7.5)	9 (11.3)	1,523,941	3	19	1 (11.1)	2 (22.2)	1 (11.1)	4 (44.4)	1 (11.1)	0 (0.0)
6	40,237,005	455	34 (7.5)	15 (44.1)	1,078,292	3	22	8 (53.3)	0 (0.0)	0 (0.0)	2 (13.3)	5 (33.3)	0 (0.0)
7	39,934,596	463	32 (6.9)	4 (12.5)	1,006,581	1	12	1 (25.0)	0 (0.0)	1 (25.0)	2 (50.0)	0 (0.0)	0 (0.0)
8	39,684,391	529	45 (8.5)	16 (36.5)	2,864,057	6	40	7 (43.8)	1 (6.3)	2 (12.5)	1 (6.3)	5 (31.3)	0 (0.0)
9	39,070,162	460	33 (7.2)	11 (33.3)	2,113,964	4	23	4 (36.4)	2 (18.2)	2 (18.2)	0 (0.0)	1 (9.1)	0 (0.0)
10	38,264,924	533	42 (7.9)	7 (16.7)	1,572,347	3	24	4 (57.1)	0 (0.0)	0 (0.0)	2 (28.6)	1 (14.3)	0 (0.0)
11	38,197,540	336	28 (8.3)	8 (28.6)	1,950,802	4	33	2 (25.0)	1 (12.5)	1 (12.5)	2 (25.0)	2 (25.0)	0 (0.0)
12	36,327,582	415	40 (9.6)	8 (20.0)	1,762,246	3	30	3 (37.5)	0 (0.0)	2 (25.0)	1 (12.5)	1 (12.5)	1 (12.5)
13	35,955,507	443	47 (10.6)	10 (21.3)	1,718,906	3	24	4 (40.0)	1 (10.0)	0 (0.0)	2 (20.0)	3 (30.0)	0 (0.0)
14	33,816,358	401	25 (6.2)	3 (12.0)	496,405	1	6	1 (33.3)	0 (0.0)	0 (0.0)	0 (0.0)	2 (66.7)	0 (0.0)
15	31,726,440	379	29 (7.7)	4 (13.8)	888,100	2	11	2 (50.0)	0 (0.0)	1 (25.0)	1 (25.0)	0 (0.0)	0 (0.0)
16	31,510,408	395	39 (9.9)	12 (30.8)	2,412,910	4	26	11 (91.7)	0 (0.0)	0 (0.0)	1 (8.3)	0 (0.0)	0 (0.0)
17	26,587,828	287	26 (9.1)	10 (38.5)	1,272,541	5	30	7 (70.0)	0 (0.0)	1 (10.0)	1 (10.0)	1 (10.0)	0 (0.0)
18	22,603,465	247	6 (2.4)	0 (0.0)	na	0	0	na	na	na	na	na	na
19	21,711,631	226	23 (10.2)	8 (34.8)	1,986,438	2	19	3 (37.5)	1 (12.5)	3 (37.5)	0 (0.0)	1 (12.5)	0 (0.0)
Total/mean	755,460,783/ 39,761,093.8	9,079	742	189 (25.5)	30,985,041/1,721,391.2	56/2.95	446	165 (43.5)	15 (7.5)	37 (12.4)	66 (19.1)	60 (15.5)	3 (2.1)
SD	11,601,527.4	165.6	15.8	2.8 (12.1)	740,239.6	1.58	13.1	18.7	13.5	10.8	16.5	16.0	7.9
Max	64,609,245	785	76	12 (44.1)	3,181,048	6	58	91.7	53.3	37.5	50.0	66.7	33.3
Min	21,711,631	226	6	0 (0.0)	496,405	0	0	11.1	0.0	0.0	0.0	0.0	0.0

Note

Geographical scenarios tested: total region (T), northern region (N), southern region (S).

We also inspected the most consistent genomic regions under divergent selection defined by the presence of three or more outlier loci in the window, showing highly significant genotypic disequilibrium (mostly *p* < 0.001) and a *r* ^2^ above the highest detected in the population analysis on average genomic windows (>0.05; Figure 2). A total of 10 genomic regions located in C1, C6 and C13 (two regions per chromosome) and C2, C3, C4 and C12 (one region per chromosome) encompassing 7.5 Mb were identified, averaging 754 kb and ranging from 427 to 1544 kb (Table S4). All these observations suggest selective sweeps at those regions, that is selection of favourable haplotypes driven by particular environmental factors determining LD and/or loss of genetic diversity. These regions included clusters of outliers (average 4.9), ranging from 3 (several) to 12 (C4 and C12) with a total of 106 annotated genes (Tables S4 and S5). Although no significant functionally enriched GO terms were identified (FDR 5%) among this set of genes, several ubiquitin‐related genes, ATP‐binding kinases, Kelch domain‐related proteins, DNA binding/metabolism‐related proteins, transporter, exocytosis and transmembrane proteins were found, four of them (ubiquitin conjugation factor E4 B, reverse transcriptase domain‐containing protein, NRAMP, monocarboxylate transporter 12‐like) including paralogous copies in the same or in different chromosomes (Table S5).

### 3.3 Population structure: temporal and geographical factors

All pairwise *F*_ST_ values between temporal replicates were nonsignificant (*p* > 0.050), suggesting temporal genetic stability between consecutive cockle cohorts in the Northeast Atlantic (Table S6). This stability was confirmed when integrating the whole data set using an AMOVA analysis, where the percentage of variation associated with differences among temporal replicates within beds (*F*_SC_) was nonsignificant and negligible, while the percentage among sampling sites (*F*_CT_) was highly significant (*p* < 0.001) and above 3% (Model I, Table 4).

**TABLE 4:** AMOVAs for European *Cerastoderma edule* beds in the Northeast Atlantic region. For models based on fastSTRUCTURE results (II, III and IV), beds were grouped following the groups found with this programme

	*F*‐statistic	Variance component	% Variation
Model I—Temporal (6 groups)			
Among beds (*F*_ST_)	0.03501***	3.58724	3.50
Among sampling sites (*F*_CT_)	0.03513***	3.59951	3.51
Among temporal replicates within sampling site (*F*_SC_)	−0.00012 NS	−0.01227	−0.01
Within beds		98.87860	96.50
Model II—STRUCTURE—all data set (*K* = 2)			
Among beds (*F*_ST_)	0.03769***	9.14631	3.77
Among groups (*F*_CT_)	0.02977***	7.22427	2.98
Among beds within groups (*F*_SC_)	0.00816***	1.92204	0.79
Within beds		233.52461	96.23
Model III—STRUCTURE—neutral data set (*K* = 3)			
Among beds (*F*_ST_)	0.01636***	2.85579	1.64
Among groups (*F*_CT_)	0.01217***	2.12470	1.22
Among beds within groups (*F*_SC_)	0.00424***	0.73109	0.42
Within beds		171.70434	98.36
Model IV—STRUCTURE—outliers (*K* = 2/*K* = 9)			
Among beds (*F*_ST_)	0.17164***/0.13253***	6.46766/4.76845	17.17/13.25
Among groups (*F*_CT_)	0.13357***/0.12288***	5.03316/4.42131	13.36/12.29
Among beds within groups (*F*_SC_)	0.04394***/0.01100***	1.43450/0.34714	3.81/0.96
Within beds		31.21308/31.21308	82.84/86.75

Note

NS, non significant (*p* > 0.05).

**p < 0.01; ***p < 0.001.

Pairwise *F*_ST_ values were significant between all the studied beds, except for some comparisons involving neighbouring sampled beds (Table S6A). *F*_ST_ for the whole data set ranged from −0.0171 (Lombos do Ulla—SLO_17 vs. Sado Estuary—PSA_19) to 0.0546 (Dee Estuary—WDE_17 vs. Sado Estuary—PSA_19), with a global *F*_ST_ value of 0.0240 (*p* < 0.001). *F*_ST_ values increased when only divergent outlier loci were considered (global *F*_ST_ = 0.1158, *p* < 0.001), ranging from −0.0044 (Lombos do Ulla—SLO_17 vs. Ria de Aveiro—PRA_17) to 0.2196 (Dee Estuary—WDE_17 vs. Sado Estuary—PSA_19), and decreased, even below the whole data set, when only neutral loci were considered (global *F*_ST_ = 0.0107, *p* < 0.001; from −0.0234 (Ria de Noia—SNO_17 vs. Sado Estuary—PSA_19) to 0.0295 (IDC_18 vs. PRF_18); see Table S6B). A consistent distribution of genetic differentiation according to geographical distance was found, confirmed by a significant isolation by distance (IBD) pattern (complete data set: *r* = 0.60214, *p* < 0.001; neutral data set: *r* = 0.42149, *p* < 0.001; outlier data set: *r* = 0.69568, *p* < 0.001; Table S7).

**FIGURE 3:**
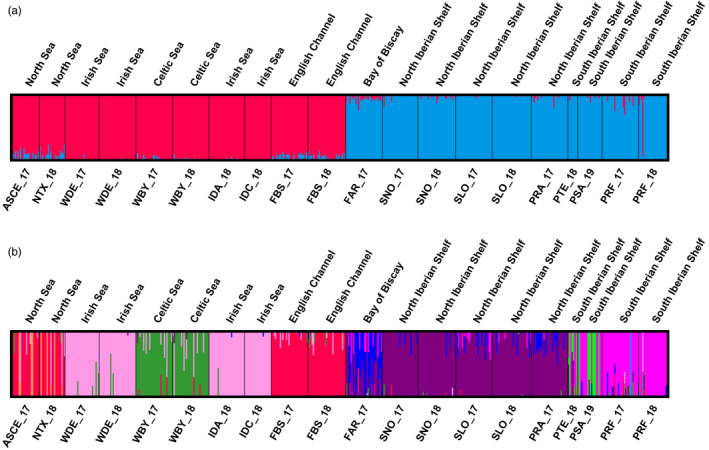
Population structure of *Cerastoderma edule* in the studied region using fastSTRUCTURE for the complete data set (a) and the divergent outlier data set (b) taking into account all beds studied. Each vertical bar represents one individual, and the colour proportion for each bar represents the posterior probability of assignment of each individual to the different clusters (*K*) inferred by the programme (*K* = 2 and *K* = 9 for a and b respectively). Codes are shown in Table 1

Bayesian clustering analysis performed with fastSTRUCTURE using the complete data set (i.e. 9309 SNPs) rendered a value of *K* = 2 as the most probable population structure (Figure 3a). One group was formed by the northern beds (above 48°N), including English Channel (Bay of Somme, France), southern North Sea (The Netherlands and Germany) and Celtic and Irish Seas, while the southern group was constituted by the beds below that parallel including the Bay of Biscay (Arcachon, France) and the western Iberian coast beds (Figure 3a). Significant IBD patterns using the different SNP data sets were suggested in the northern group (complete data set: *r* = 0.49761, *p* = 0.041; neutral data set: *r* = 0.35403, *p* = 0.074; outlier data set: *r* = 0.65359, *p* = 0.020) and southern group (complete data set: *r* = 0.44919, *p* = 0.067; neutral data set: *r* = 0.55559, *p* = 0.039; outlier data set: *r* = 0.58677, *p* = 0.003), indicating that the IBD pattern observed in the whole Northeast Atlantic region is not an artificial consequence of vicariance between two genetically differentiated genetic groups. AMOVA using these two groups showed that this structuring (*F*_CT_ = 2.98% of the total genetic diversity) captured close to the 80% of the total differentiation among beds (*F*_ST_ = 3.77%) (Model II, Table 4). The best *K* values for the neutral and the outlier data sets were 1 and 2, respectively, with identical results to those found with the complete data set when the number of simulated clusters was low (3 and 2, respectively; Figure S2). AMOVA results with the neutral data set were similar to those obtained with the whole data set, the main difference being also detected among groups (*F*_CT_ = 1.22%) close to 75% of total differentiation among beds (Model III, Table 4). The second most probable clustering suggested by the outlier data set (*K* = 9) identified six main well defined groups: (i) North Sea and English Channel beds up to the Bay of Somme (ASCE_17, NTX_18, FBS); (ii) the Dee bed in North Wales and Irish beds in the Irish Sea (WDE, IDA_18 and IDC_18); (iii) the Burry bed in South Wales in the Celtic Sea (WBY); (iv) the Arcachon bed in the Bay of Biscay (FAR_17); (v) the Northwest Iberian coast including Spanish and the northern Portuguese beds (SNO, SLO, PRA_17); (vi) the Southwest Iberian beds (PTE_18, PSA_19, PRF) (Figure 3b). AMOVAs with the outlier data set and these nine groups assigned the highest percentage of genetic variation to differences among groups (92.8% of the variation among beds) in comparison with the whole and neutral data sets (Model IV, Table 4), while the lowest variance proportion among beds within groups (Table 4), confirming their genetic homogeneity. Within the two main northern and southern groups, fastSTRUCTURE suggested *K* = 1 as the most probable value for all the analyses except for those using outlier data sets. With these latter data sets, the most likely structure of the northern and southern groups was identical to that suggested by the analysis with the complete data set (three groups in the northern region and three in the southern region, see Figure S2). Moreover, for the northern region, at higher number of simulated clusters (*K* = 7) each bed constituted a single cluster (Figure S2). Although sampling with higher spatial resolution in the Bay of Biscay could aid to refine genetic structure in edible cockle, ours results suggest that the cluster structure found is consistent.

**FIGURE 4:**
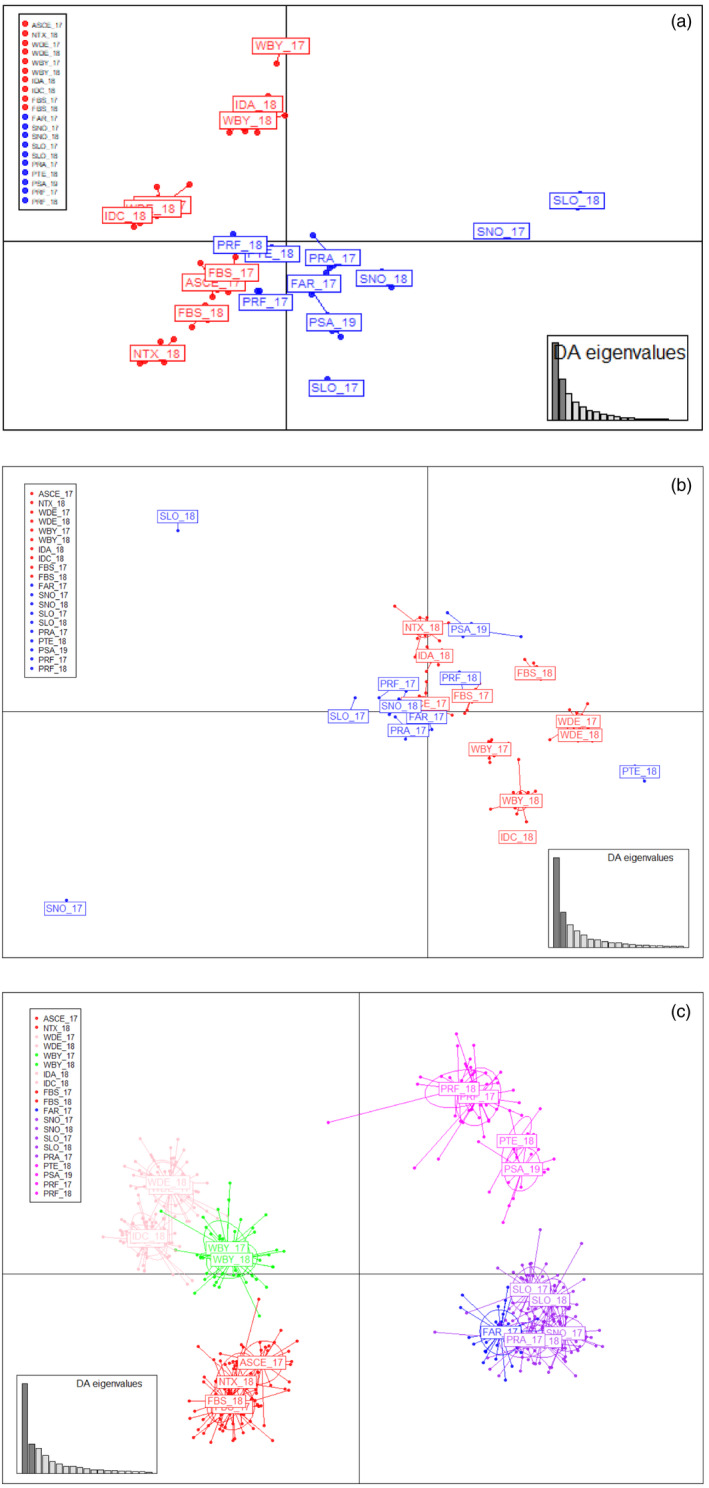
Discriminant analysis of principal components plots of *Cerastoderma edule*. The weight of retained discriminant analysis (DA) eigenvalues representing >90% of variance are shown on right bottom box. Results using the complete data set (a), neutral data set (b) and divergent outlier data set (c) are shown. Codes are given in Table 1

Discriminant analyses of principal components plots confirmed the results found with fastSTRUCTURE regarding the main north‐south subdivision, but further clustering was suggested within groups. The analyses with the complete and neutral data sets showed an important scattering within each group (Figure 4a,b), while the analysis with the outlier data set clearly identified four main differentiated groups: (i) North Sea and English Channel (ASCE_17, NTX_18, FBS); (ii) Irish and Celtic seas (WDE, IDA_18, IDC_19 and WBY); (iii) the Bay of Biscay and north Iberian waters (FAR_17, SNO, SLO and PRA_17); and (iv) south Iberian waters (PTE_18, PSA_19, PRF). A more subtle subdivision with up to the six groups observed with fastSTRUCTURE can be distinguished using outlier loci (Figure 4c). Finally, DAPCs within northern and southern groups using their respective outlier data sets also fitted with the clustering suggested by fastSTRUCTURE (Figure S3).

**FIGURE 5:**
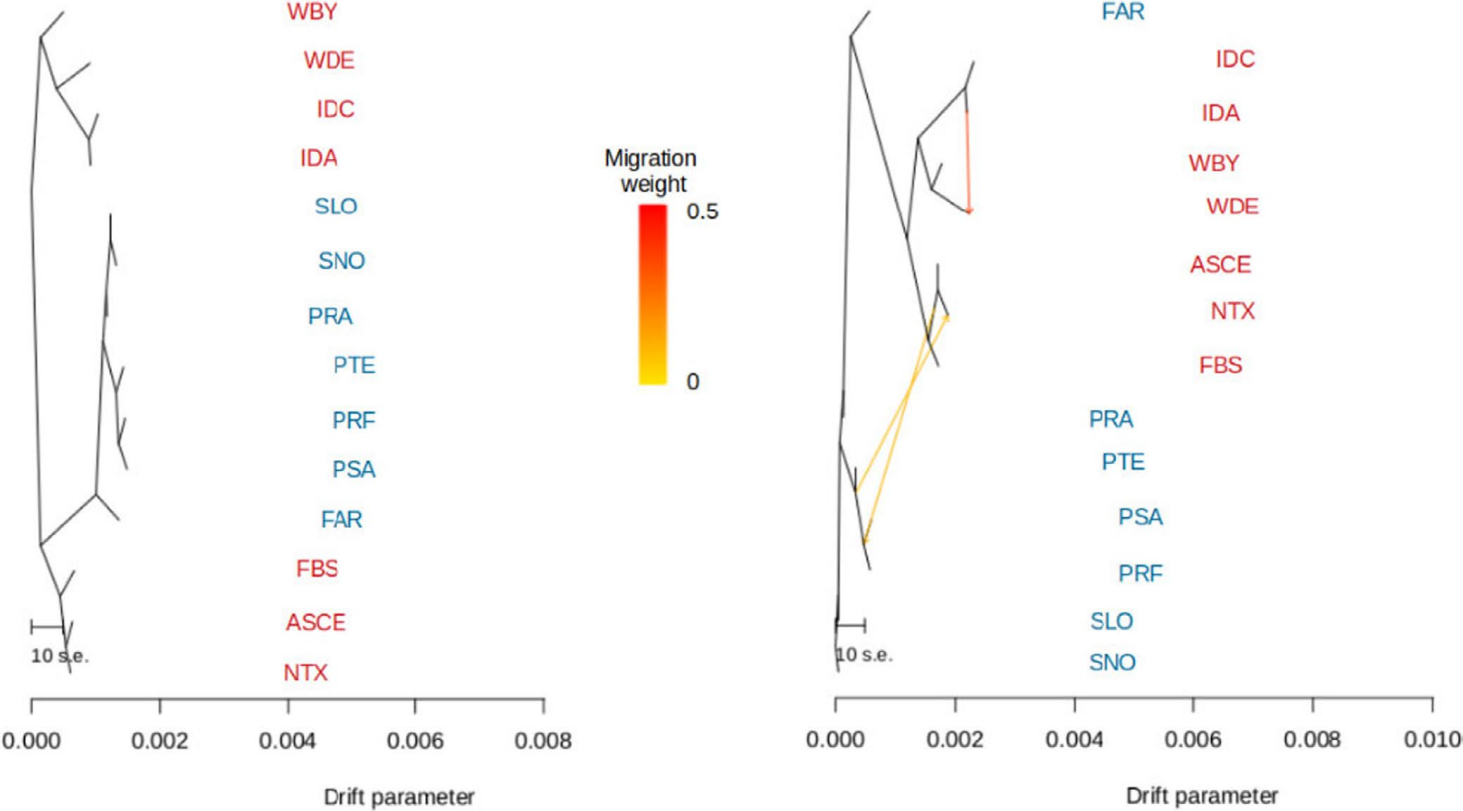
Maximum‐likelihood trees inferred by TREEMIX without (left) and with (right) migration events included. Migration events are depicted as heatmap coloured arrows from yellow to red. Population code colours: northern region (red), southern region (blue)

The population trees provided by TREEMIX without migration events also identified the aforementioned groups, clustering beds according to northern and southern groups and identifying the substructure suggested by outliers (Figure 5). The best number of migration events was three, with the strongest connection between Irish Sea beds and weaker connections between beds from the northern and the southern groups. An additional TREEMIX analysis was also run with beds grouped according to the six previously described clusters identified with fast STRUCTURE based on 554 outlier markers. This tree provided similar results with the clustering of i, ii, iii (northern group) in one branch and iv, v and vi (southern group) in the other. The best fitting model was for three migration events, with the connection between groups i and iv being strongest (Figure S4).

### 3.4 Genetic–environmental associations

**FIGURE 6:**
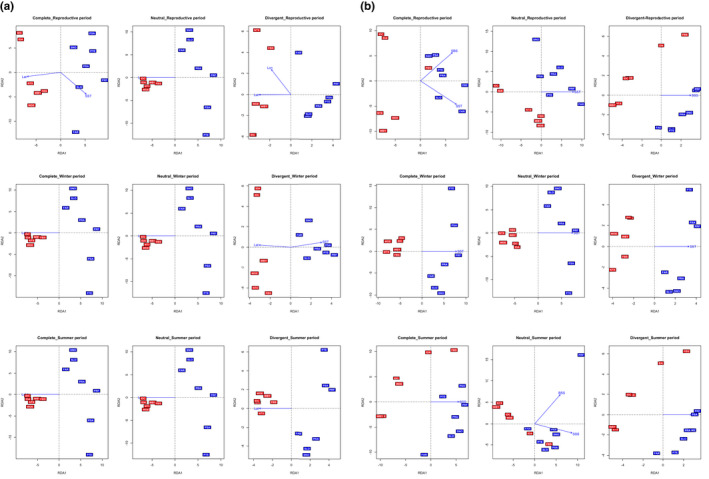
Redundancy analyses (RDA) plots of *Cerastoderma edule* samples from the studied area using the different genetic data sets (complete: complete data set; neutral: neutral data set; divergent: divergent outlier data set) and seasons (reproductive period, winter and summer) taking into account all landscape variables (a) and only abiotic factors (b). BSS, bottom shear stress; SBS, sea bottom salinity; SSS, sea surface salinity; SST, sea surface temperature. Population code colours: northern region (red), southern region (blue)

When all the seascape variables were included in the RDA analyses, latitude was suggested as the main driver for genetic differentiation, for the three different data sets (i.e. complete, neutral and outlier divergent loci) and seasons (i.e. reproductive, winter and summer) analysed (Table 5). Latitude was mainly associated with the first axis (Figure 6a), which separated the beds in the two groups suggested by fastSTRUCTURE (i.e. northern and southern groups). Moreover, sea surface temperature (SST) was a suggested driver for the complete and divergent data sets during the reproductive and winter periods respectively. When spatial variables (latitude and longitude) were excluded from the analysis, SST was the main driver in most scenarios, separating northern and southern groups for all data sets (Figure 6b). Also, SSS appeared as the main driver during summer for all data sets and in the reproductive period for the outlier data set (Table 5). For this driver, the north‐south separation was not as clear and some intermingling could be observed especially considering the horizontal axis that related to SSS. However, VIF values associated to the variables suggest collinearity for many of the models studied (VIF > 5), although an important proportion showed moderate (VIF < 10) or no problems (VIF < 5, i.e. longitude); so despite this information still being useful, it should be considered with caution for interpretation and management decisions.

**TABLE 5:** Results of the redundancy analysis (RDA) on *Cerastoderma edule* beds. Only variables included by the forward selection model are shown

Model	Season	Variable	Complete data set (9309 SNPs)		8021 neutral data set		554 divergent outlier data set	
			*p*‐Value	Adjusted *R* ^2^	*p*‐Value	Adjusted *R* ^2^	*p*‐Value	Adjusted *R* ^2^
All seascape variables	Reproductive period	Latitude	0.001	0.092	0.002	0.075	0.001	0.332
		Longitude	—		—		0.139	
		SST	0.023		—		—	
	Winter	Latitude	0.001	0.088	0.001	0.075	0.001	0.331
		SST	—		—		0182	
	Summer	Latitude	0.001	0.088	0.001	0.075	0.001	0.304
Only abiotic variables	Reproductive period	SST	0.003	0.072	0.004	0.045	—	0.159
		SSS	—		—		0.010	
		SBS	0.087		—		—	
	Winter	SST	0.002	0.084	0.002	0.072	0.002	0.298
	Summer	SSS	0.014	0.050	0.036	0.051	0.005	0.171
		BSS	—		0.002		—	

Note

Adjusted *R* ^2^ and *p*‐value associated to each variable of its selection stage.

Abbreviations: BSS, bottom shear stress; SBS, sea bottom salinity; SSS, sea surface salinity; SST, sea surface temperature.

### 3.5 Current modelling and larval dispersal

**FIGURE 7:**
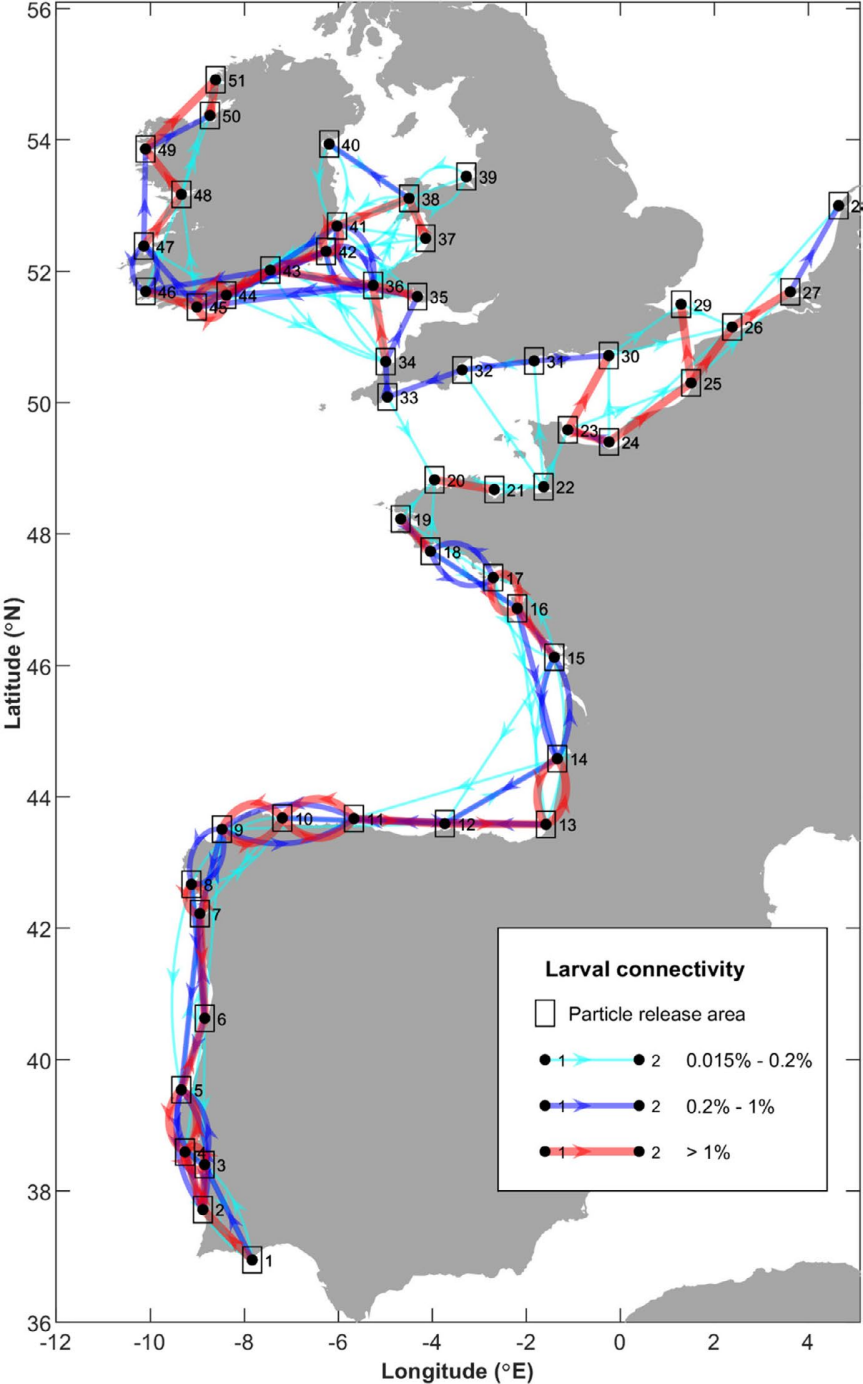
Mean larval connectivity pathways for April to September from 2016 to 2018 releases at depths of 1, 15 and 30 m. The direction of the arrows indicates the direction of larval transport, and the colour and thickness of the connection display the strength of the connection

Connectivity pathways from the larval dispersal modelling, time‐averaged and averaged across all three depth scenarios are shown in Figure 7 and Figure S5. All sites along the West Iberian Shelf, the Cantabrian Sea and the Bay of Biscay up to French Brittany (sites 1–19) were simulated to be potentially well connected to multiple neighbouring sites along the coastline with relatively high levels of connectivity (>1%). Even if simulated connectivity between neighbouring sites was <1%, generally multiple connection pathways were simulated. Across the headland of French Brittany into the English Channel, where genetic differentiation was established, the modelling simulated weak connectivities (<0.2% between sites 18/19 and site 20, on average, with several scenarios simulating no connectivity). Sites in the eastern part of the English Channel and along the north coast of the channel were well connected with each other. The sites (20–22) along the north coast of Brittany were less well connected with the other areas of the channel; however, multiple connections did exist between sites 20–22 and sites along the northern part of the channel and the eastern part of the channel, suggesting that while this area may be more isolated, it is probably not fully isolated from the other parts of the channel. Similar to French Brittany, we simulated relatively weak (and one‐way only) connectivity from the English Channel to the Celtic Sea, around the headland of southwest Britain (connecting site 33–34). Sites within the Celtic Sea were simulated to be well connected: >1% between many sites and with notable westward larval transport along the Celtic Sea Front potentially connecting sites in the eastern Celtic Sea (e.g. site 36–43) with the sites along the south coast of Ireland. Along the south and west coast of Ireland, particles became entrained in the Irish Coastal Current and were transported westwards then northwards along the coast, thus simulating potentially strong connectivities between sites 41 and 51.

Taken individually, the larval behaviour scenarios (i.e. particle cohorts released (and fixed) at 1, 15 and 30 m water depths) showed considerable differences in the simulated connectivity networks. The surface (1 m) release scenario (Figure S6) simulated the highest degree of connectivity with almost all sites receiving larvae from at least two other nearby sites and sites being interlinked along most coast lines. The 15 m scenario (Figure S7) simulated a similar picture, with a well‐connected Iberian and Bay of Biscay coastline in particular, and a well‐connected Irish and Celtic Sea region. However, simulated connections from the French Atlantic coast into the English Channel and from the English Channel into the Celtic Sea were much weaker (<0.2%, on average, with periods of no connectivity). In the English Channel, the eastern sites remained well connected, apart from site 28. Only weak connections were seen between the northern and southern sites in the western part of the channel, effectively splitting the western channel into a northern and a southern group. Connections between the eastern and western part of the channel were also limited to a weak connection between sites 22 and 23, thus also isolating the eastern and western parts of the channel from each other. For the 30 m scenario (Figure S8), we simulated the same areas to be well‐connected, but no dispersal was simulated between southern and northern French Brittany, nor between southern and northern Cornwall, thus isolating the Atlantic sites, the English Channel and the Celtic/Irish Sea sites from each other. In the English Channel, the eastern part of the channel remained relatively well connected but, in the west, connectivities between sites (apart from sites 20 and 21) and across the channel were weak, and as for the 15 m depth scenario, the connectivities between the eastern and the western sites in the channel were weak. However, when the 15 and 30 m scenarios are considered for months June and July only (Figure S9), connectivities in the English Channel were much stronger. While connectivities were weak between the sites 31 and 32, site 32 also received larvae from 20 to 23, thus indicating that, during the middle of the spawning season of *C*. *edule*, the eastern and western (and northern and southern) areas of the channel are connected, albeit weakly, suggesting that sites in the channel may be grouped into one unit.

The simulated interannual variations in connectivities were greatest for the surface release scenario and decreased the deeper the larvae were in the water column (results not shown). For the 15 and 30 m depth scenarios, simulated connectives over different years tended to vary in strength but much less so in overall patterns. Overall, the larval dispersal modelling suggested that the sites may be grouped into three distinct cohorts: (i) the Iberian and Bay of Biscay Atlantic coast sites; (ii) the English Channel sites; and (iii) and the Celtic and Irish Sea sites.

## 4 DISCUSSION

### 4.1 Genetic diversity

Genetic diversity is crucial for the adaptation of natural populations to environmental changes. The preservation of connectivity among populations is essential to maintain the genetic diversity within and between populations thus contributing to their genetic robustness and aiding to counteract the threat of demographic depletion caused by overexploitation or diseases (Frankham et al., 2002). Moreover, genetic diversity in wild populations is important for commercial species, such as *C*. *edule*, to support breeding programmes for selecting high‐value seed for intensive production in particular areas (do Prado et al., 2018; Lallias et al., 2010; Petereit et al., 2018; Vera et al., 2019). Genetic diversity was similar across all cockle beds studied in the Northeast Atlantic (*H*_e_ range: 0.077–0.086). The genetic diversity range widened (0.153–0.290), when only polymorphic markers were considered (MAC ≥2, representing MAF >0.01 in our sample scenario), but mostly due to the Tejo Estuary representing an outlier population with much higher *H*_e_ than the rest (maximum *H*_e_ = 0.193 without Tejo Estuary). All samples showed a consistent slight heterozygote deficit (*F*_IS_ > 0), although nonsignificant, likely related to a technical genotyping issue associated with the presence of null alleles as previously reported in molluscs (Pino‐Querido et al., 2015).

As indicated above, all polymorphic loci in each bed showed MAF ≥0.01, a filter commonly used in RADseq studies, which allows the comparison of genetic diversity with previous studies. Heterozygosity figures in our study were similar to those reported by Coscia et al. (2020) (*H*_e_: 0.144–0.156), who applied the same MAF filter to characterize genetic diversity in *C*. *edule* beds from the Celtic and Irish Seas (i.e. southeast Ireland and Wales) using a classical RADseq technique (Etter et al., 2011). Genetic diversity observed for *C*. *edule* in our study was also similar to that reported for the sister species *C*. *glaucum* (MAF = 0.01; *H*_e_: 0.078–0.137; Sromek et al., 2019); however, both cockle species showed lower genetic diversity at genomic scale than other bivalves such as *Crassostrea virginica* (MAF = 0.01; *H*_e_ ~ 0.300; Bernatchez et al., 2019) *Placopecten magellanicus* (MAF = 0.05; *H*_e_ = 0.271 ± 0.133, van Wyngaarden et al., 2017), *Ostrea edulis* (no MAF filtering; *H*_e_ ~ 0.300; Vera et al., 2019) and *Crassostrea gigas* (no MAF filtering but mean MAF of selected SNPs = 0.182; *H*_e_ ~ 0.300; Gutierrez et al., 2017), although in the latter cases comparison is not so straightforward since the filtering scenario was not the same. These data suggest that despite genetic diversity differences could in part be due to filtering protocols and genotyping with preselected SNP chips, the genus *Cerastoderma* appears to lie in the lower range of genetic diversity of molluscs studied to date.

Regarding genetic diversity within the edible cockle, higher genetic diversity would be expected at lower latitudes of the Northern Hemisphere because of their role as glacial refugia during Quaternary glaciation (Hewitt, 2000). This hypothesis fits to our data as genetic diversity (*H*_e_) and the proportion of polymorphic loci (MAC ≥2) were slightly higher but significant or marginally significant in the southern group (0.803 vs. 0.798, Mann–Whitney *U* test, *p* = 0.029; 3821 vs. 3090, Mann–Whitney *U* test, *p* = 0.052). Previous studies with microsatellite loci did not detect differences between both groups (Martínez et al., 2013), and even supported higher diversity in the Celtic/Irish Seas, English Channel and the North Sea (Martínez et al., 2015) corresponding to the present northern group. Moreover, higher genetic diversity has been found in northern beds from the Fennoscandian region and Russia, using mtDNA markers, suggesting a cryptic northern glacial refuge for *C*. *edule* (Krakau et al., 2012; Martínez et al., 2015), which has also been described for other marine species (Luttikhuizen et al., 2008; Maggs et al., 2008; Sotelo et al., 2020). Our extensive genome‐wide analysis indicates slight differences in genetic diversity for the edible cockle in the whole Northeast Atlantic compatible with the existence of important glacial refuges in the south.

### 4.2 Population structure and connectivity

Knowledge of population structure, including local adaptations and historical processes, is crucial to define and apply sustainable strategies for the management and conservation of exploited species (Bernatchez et al., 2017; Frankham et al., 2002). For *C*. *edule*, the presence of at least two main population units within the Northeast Atlantic, delimited by the western English Channel, had been reported in previous studies (Krakau et al., 2012; Martínez et al., 2015) and has been confirmed in this study with a more in depth population genomic analysis using both whole, neutral and outlier SNP data sets with several complementary statistical approaches (i.e. STRUCTURE, DAPC, TREEMIX analyses). These two units would correspond to the two glacial refugia of the species during the Pleistocene, with Ireland, Great Britain and southern North Sea being the suggested area for the secondary contact between those divergent lineages (Martínez et al., 2015). The northern group in our study, comprising of the Celtic and Irish Seas, the English Channel and North Sea beds, corresponds to the northern group defined by Martínez et al. (2015). This structure has also been documented in other mollusc species with a similar distribution range such as *O*. *edulis* (Vera et al., 2016). The results from our larval dispersal modelling, based on potential ocean‐driven larval flows between sites along the Northeast Atlantic, suggest a potential discontinuity between the Bay of Biscay and the English Channel. The larval dispersal model indicates that the break in dispersal may be located at the French Brittany headland, indicating that the oceanography of the Northeast Atlantic may play an important role in shaping the genetic structure of cockles. The frontal systems (Ushant Front and Celtic Sea Front) when fully established seem to represent barriers to dispersal, thus limiting, for example, the transport of larvae into the English Channel and into the Irish Sea respectively. Headlands, such as Brittany, Cornwall and the Cotentin Peninsula, also appear to act as barriers, potentially due to diverging current systems. However, due to lack of genetic samples in close proximity on either side of these oceanographic and topographic features, this hypothesis will need to be confirmed with new genetic data in the future. Dispersal patterns within the English Channel show a considerable amount of variability between scenarios with the surface release scenario indicating a much higher degree of connectivity between sites than the scenarios with larvae lower in the water column. These scenarios highlight a potential split between populations in the east and the western parts of the channel. However, when only mid‐summer larval transport is considered no split is present, thus highlighting the importance of accurate knowledge of the timings of the main spawning events. Future research using strategic sampling of cockle beds on the shores of the channel should evaluate genetic differentiation within the English Channel to assess whether the populations in the channel can be grouped into a single unit or whether subgroups within the channel may exist.

Genomic scans help detect footprints of selection with regards to the neutral background, which represents invaluable information for sustainable management and conservation of fisheries (Bernatchez, 2016; Nielsen et al., 2009). Genetic markers showing a significant departure, above or below the neutral data set are potential outliers under divergent or stabilizing selection, respectively. Divergent outliers can unravel a fine‐scale structure related to environmental variables critical for resources management (Coscia et al., 2020; Longo et al., 2020; Schulze et al., 2020; Vandamme et al., 2021; Vera et al., 2019; Whitaker et al., 2020). Temperature, salinity and other abiotic factors have been suggested as potential drivers for adaptive differentiation in *C*. *edule* (Coscia et al., 2020), as in other marine species in the region (do Prado et al., 2018; Vera et al., 2016). In our study, the RDA analyses also suggested the effect of temperature and salinity in a wider geographical range, the major genetic subdivision north‐south in the Northeast Atlantic being associated with a latitudinal annual mean temperature gradient of ~5.5°C between the warmest (Ria de Formosa) and the coldest station (Dundalk). However, these results should be taken with caution due to the collinearity between variables in half of the scenarios tested (VIF > 10).

We carried out detailed gene mining on the most consistent genomic regions putatively under divergent selection. They were targeted using different criteria to avoid false positives (two or more consecutive outliers criterion) and to confirm characteristics compatible with selective sweeps (significance and magnitude of linkage disequilibrium). Signals of balancing selection were very weak, as expected in a low structure scenario, and the few markers detected did not meet the criteria established. The percentage of divergent outlier loci detected considering all scenarios (8.7%) was in the range reported in other studies (do Prado et al., 2018; Vera et al., 2019). Among these, 189 (2.1%) constituted groups of consecutive outliers and 59 (0.6%) fulfilled the strictest criteria: significant outlier pairwise genotypic disequilibrium (mostly *p* < 0.001) and *r* ^2^ > 0.05, which represents the highest average LD in genomic windows across the whole genome estimated for Northeast Atlantic. Two of these regions under divergent selection in C1 and C4 included 8 and 12 outliers, respectively, *r* ^2^ > 0.20 and encompassed more than 1 Mb, suggesting strong selective sweeps. Gene mining in the most consistent genomic regions identified 105 candidate genes using the incomplete transcriptome annotation carried out within the COCKLES project (9076 genes; B. G. Pardo, C. Fernández, P. Martínez, unpublished data). Groups of these genes and families were associated with relevant functions, but no functional enrichment GO terms were identified likely due to the small number of genes, poor functional annotation and the putative diverse drivers acting on the different genomic regions. More refined information should be obtained when the annotation of the cockle's genome is improved.

A refined analysis with outlier loci enabled us to disclose a significant substructure in the Northeast Atlantic beyond the two major groups identified with all data sets as outlined above. Thus, the northern group would be split into three major subgroups with important connections as suggested by TREEMIX analysis including the Irish Sea beds, the Celtic Sea beds (both in the west), and the English Channel and North Sea beds in the east, also interconnected. Concurrently, this northern subdivision was also detected with the larval dispersal modelling performed here. Within the southern group, three subgroups would also be differentiated: southern Iberian Shelf (where PTE_18 is also differentiated from PSA_19 and PRF), northern Iberian Shelf (PRA_17, SNO, SLO) and Arcachon in the central Bay of Biscay (FAR_17). In the larval dispersal modelling, weaker connectivity was also observed between southern and northern Portuguese subgroups during June and July at depths of 15 and 30 m (Figures S9 and S10), in contrast with the strong connections between the other release locations along the Iberian coastline, but more data on the spawning period are needed to confirm the role of ocean currents on the observed genetic subdivision (Mahony et al., 2020). In fact, strong connectivities were detected during the other simulated months. Northwards, Cape Finisterre has been suggested as a biogeographical barrier for marine organisms (Abaunza et al., 2008; López‐Jamar et al., 1992; Piñeira et al., 2008). Unfortunately, the beds along the southern Bay of Biscay were not analysed in our study, but the Arcachon bed (FAR_17) in the southeast Bay of Biscay was differentiated from the northern Iberian Shelf samples, suggesting a possible genetic break between the north‐western Iberian Shelf and the Bay of Biscay coast. Martínez et al. (2013) suggested Cape Finisterre as a biogeographical barrier for *C*. *edule* (separating northern Iberian beds from those in the Bay of Biscay) but, as in the present study, beds from the southern Bay of Biscay were not included. Holey sampling in this region prevents sound seascape conclusions about the putative break responsible for the observed differentiation (Riginos et al., 2016). Moreover, Chust et al. (2013) did not find genetic differentiation in *C*. *edule* between this region and north‐western Iberian beds using microsatellites, suggesting that Cape Finisterre would not appear to represent a barrier to gene flow as found in other species (Domingues et al., 2010; López et al., 2015; Riquet et al., 2019). This substructure did not appear to be related to dispersal limitations of larvae according to our modelling data and neutral data set. Therefore, more detailed sampling should be carried out in the Bay of Biscay to define the potential biogeographical barriers in this region. Northwards, the areas in the central Bay of Biscay defined as the ICES VIIIb fishery subzone (Bay of Biscay centre) have previously been described as potential barriers for different marine organisms such as *Hippocampus guttulatus* (Riquet et al., 2019) and *Zoostera noltei* (Chust et al., 2013).

As outlined above, the genetic structure of the edible cockle in the Northeast Atlantic shows a notable correspondence with the larval dispersal modelling performed here, although some discordances are suggested depending on the set of markers used (neutral vs. outlier loci). However, both data sets might be fully compatible if other variables such as larval depth in the water column is considered in larval dispersal modelling. The simulations carried out with larvae located at three different depths suggest that deeper depths (15 and 30 m) correlate better with the major subdivision observed with neutral loci (north vs. south) and are in accordance with other studies on bivalve larval behaviour, which show that larvae are preferably located below the surface of the water column rather than close to it (Irigoien et al., 2004; Scrope‐Howe & Jones, 1986). This result also highlights the importance of oceanographic studies of larval distributions throughout the water column, and that this knowledge on larval behaviour cannot be solely gained from tank experiments under laboratory conditions. Large uncertainties persist since the larval behaviour of *C*. *edule* with regard to swimming behaviour remains to be studied in detail. While the surface releases showed a higher degree of interannual variability, the deeper releases had stable connectivity patterns between years, thus tying in with the genetic structure which seems stable interannually.

It could be expected that larval dispersal and environmental variables had distinct imprints on genetic diversity, unless patterns of dispersal and environmental variables were correlated and influenced by the same oceanographic features. Moreover, different spatial patterns of genetic diversity associated with neutral and outlier markers would be expected, the former being related to ocean current patterns and fronts and outlier loci with environmental factors driving selection (Riginos et al., 2016). However, as suggested before, a correlation between larval dispersal and environmental factors seems to occur in the northeast Atlantic scenario, and thus, neutral and outlier markers show quite similar outcomes at least at larger scales. This is clear when considering the two groups divided at French Brittany and the related north–south variation in temperature, but it is likely that ocean current patterns and seasonally generated frontal flows may too be structuring environmental ecosystems, and hence the correlation observed, not only at macrogeographic level, but also at a meso‐scale. Moreover, both types of markers would also be affected by historical population processes, and accordingly, outliers could also be disclosing other phenomena different from selection, such as hybridization between the main genetic units identified or secondary contacts between divergent lineages related to glacial refugia. Thus, genomic regions associated with divergent selection could be related to local adaptation, but also to genetic incompatibilities or differences in mate choice among differentiated units or lineages (Bierne et al., 2011, 2013; Vilas et al., 2015). A refined analysis of genetic diversity at a local scale around the main fronts and physical barriers described here additionally covering holy sampling in the Biscay Bay would be essential to disentangle the different factors shaping genetic variation of the edible cockle in the Northeast Atlantic.

### 4.3 Fisheries management

Despite sampling limitations (e.g. limited sampling along Bay of Biscay), our results provide useful information for the management of cockle beds in the Northeast Atlantic and could be valuable for obtaining suitable seed either for restocking of depleted populations or for finding broodstock to enhance cockle production. An improved definition of management units considering both demography and adaptation to environmental variation along the Northeast Atlantic could be initially delineated, allowing the possible future definition of adaptive management units (AMU, Bernatchez et al., 2017). Two main operational units, located northwards and southwards French Brittany, might be defined as the basic proposal for management in the Northeast Atlantic region, but a more detailed approach could include at least six different units: (i) Irish Sea; (ii) Celtic Sea; (iii) English Channel/North Sea; (iv) Bay of Biscay; (v) northern Iberian Shelf; and (vi) southern Iberian Shelf. More refined analysis at microgeographic level around the discontinuities together with further information on larval behaviour and more realistic modelling would aid to a more detailed picture. Further, the imminent release of the annotated cockle's genome will add more refined information on candidate genes in genomic regions subjected to divergent selection to detect signals of local adaptation. Moreover, the information from this study might be useful to define sets of markers, starting from outlier loci, which could be applied to found brookstock for restocking depleted populations and to track individuals to their units that could aid the identification of illegal transferences between countries or from disease‐affected areas. Our data represent the baseline to monitor restocking and to evaluate the impact of intensive aquaculture on cockle beds.

## CONFLICT OF INTEREST

Authors have no conflict of interest to declare.

## Supporting information

Fig S1‐S10

Table S1‐S7

## Data Availability

Data for this study are available at Dryad Digital Repository (https://doi.org/10.5061/dryad.kh189326p) and Supporting Information
