# Supplementary material for: Genomic survey of edible cockle (Cerastoderma edule) in the Northeast Atlantic: A baseline for sustainable management of its wild resources
Source: Evol Appl. 2022 Jan 25;15(2):262–85. doi: 10.1111/eva.13340 (PMC8867702; doi:10.1111/eva.13340)
Supplement: Supplementary file 1 — Fig S1‐S10 [file EVA-15-262-s001.docx]

| A  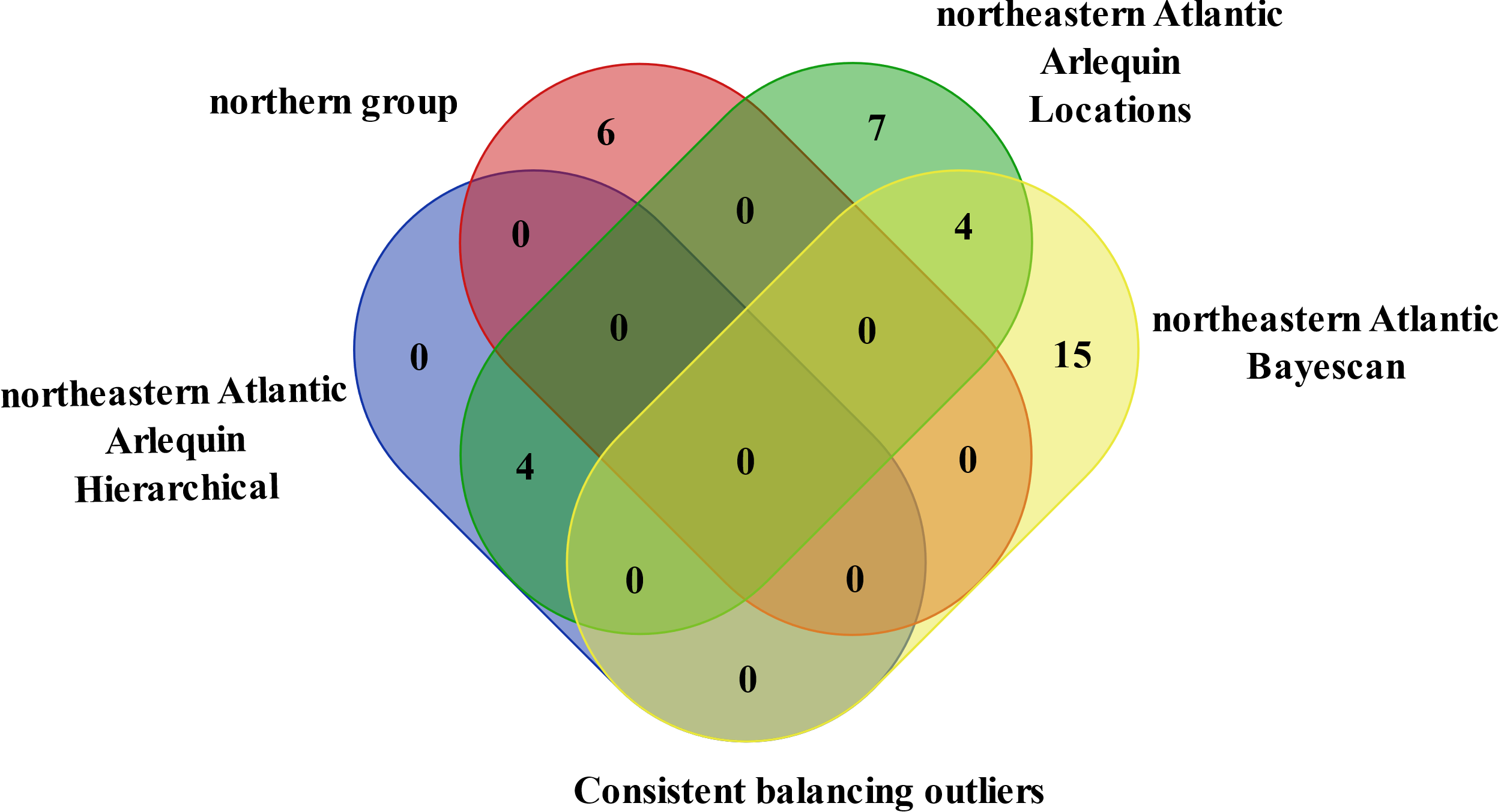 |
| --- |
| B  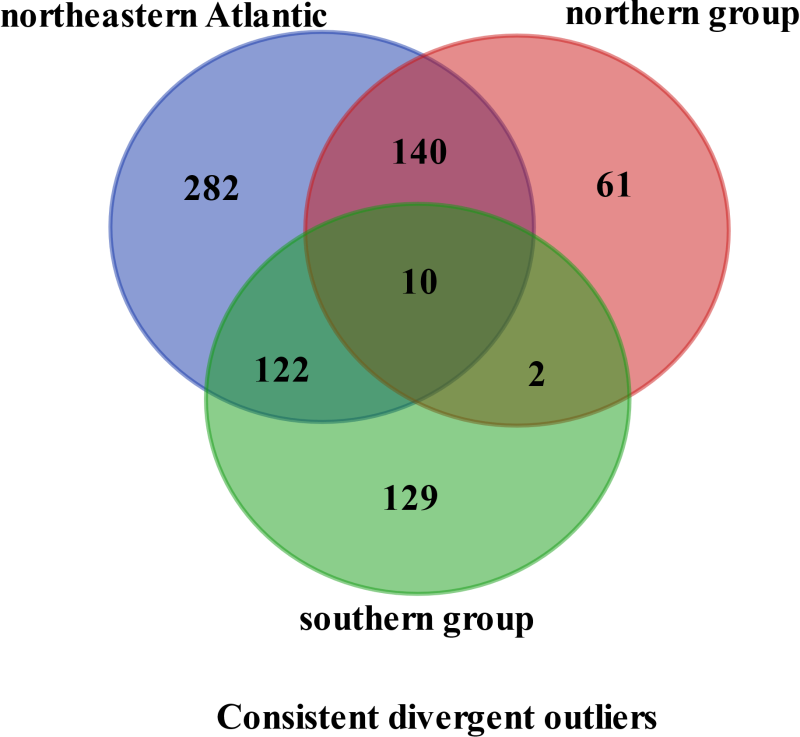 |

**Supplementary Figure 1.** Venn diagrams for consistent outliers. Different groups are defined on Table outliers.

| A   |
| --- |
| B   |
| C   |
| D   |
| E   |

**Supplementary Figure 2.** Population structure of *Cerastoderma edule* in the northeast Atlantic using fastSTRUCTURE for: (A) K =3 with the neutral dataset and all the beds; (B) K = 2 with divergent outlier dataset and all the beds; (C) K = 3 with de divergent outlier dataset in the northern group; (D) K = 7 with de divergent outlier dataset in the northern group; and (E) K = 8 with de divergent outlier dataset in the southern group. Each vertical bar represents one individual, and the colour proportion for each bar represents the posterior probability of assignment of each individual to the different clusters (K) inferred by the program. Codes are shown on Table 1.

| A- Divergent outlier dataset northern group  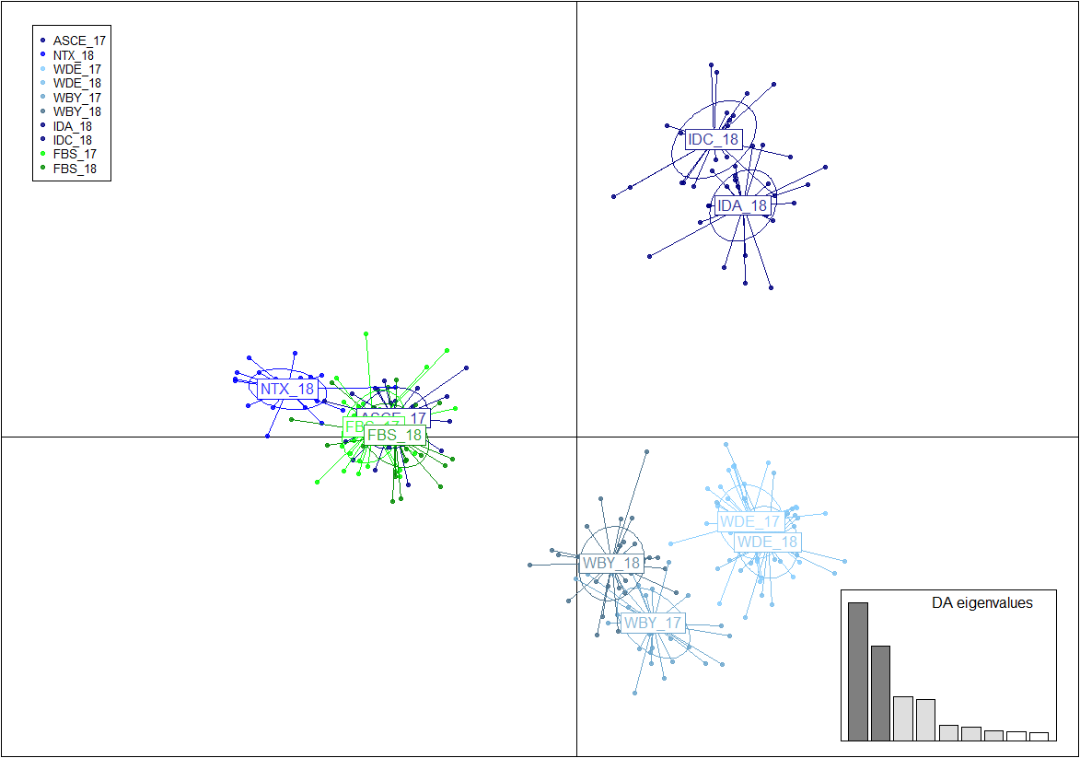 |
| --- |
| B- Divergent outlier dataset southern group  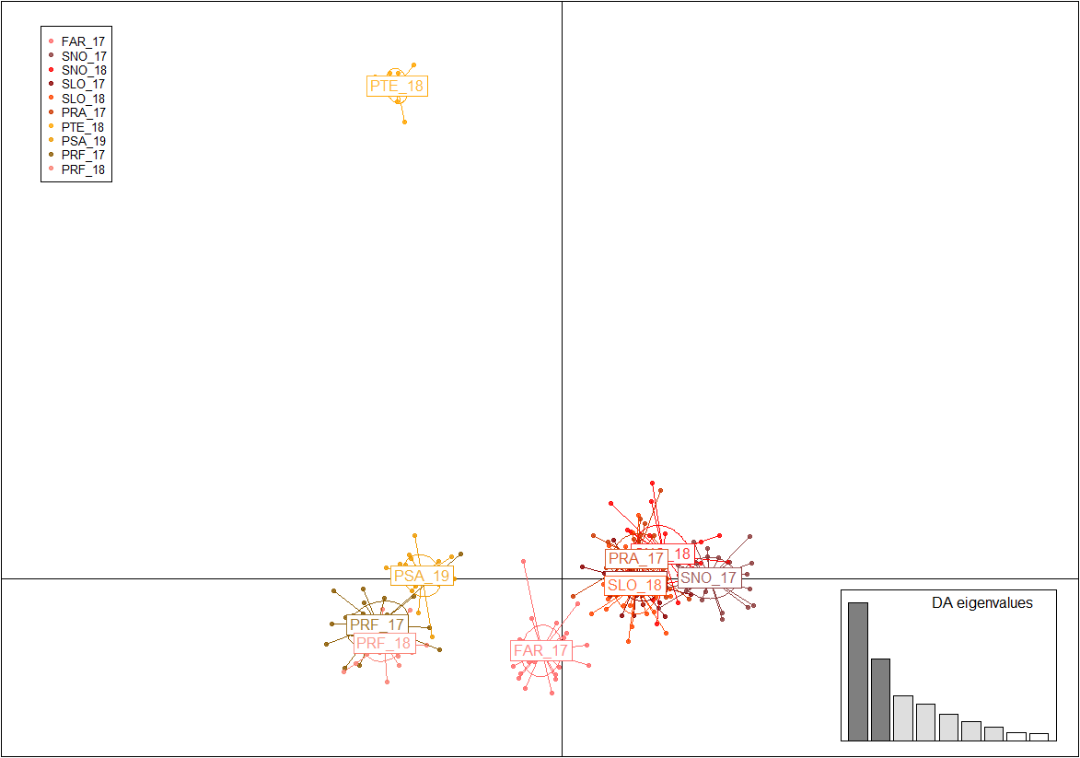 |

**Supplementary Figure 3.** DAPCs plots for northern (A) and southern (B) groups using their respective divergent outlier dataset. Bed codes are shown on Table 1.


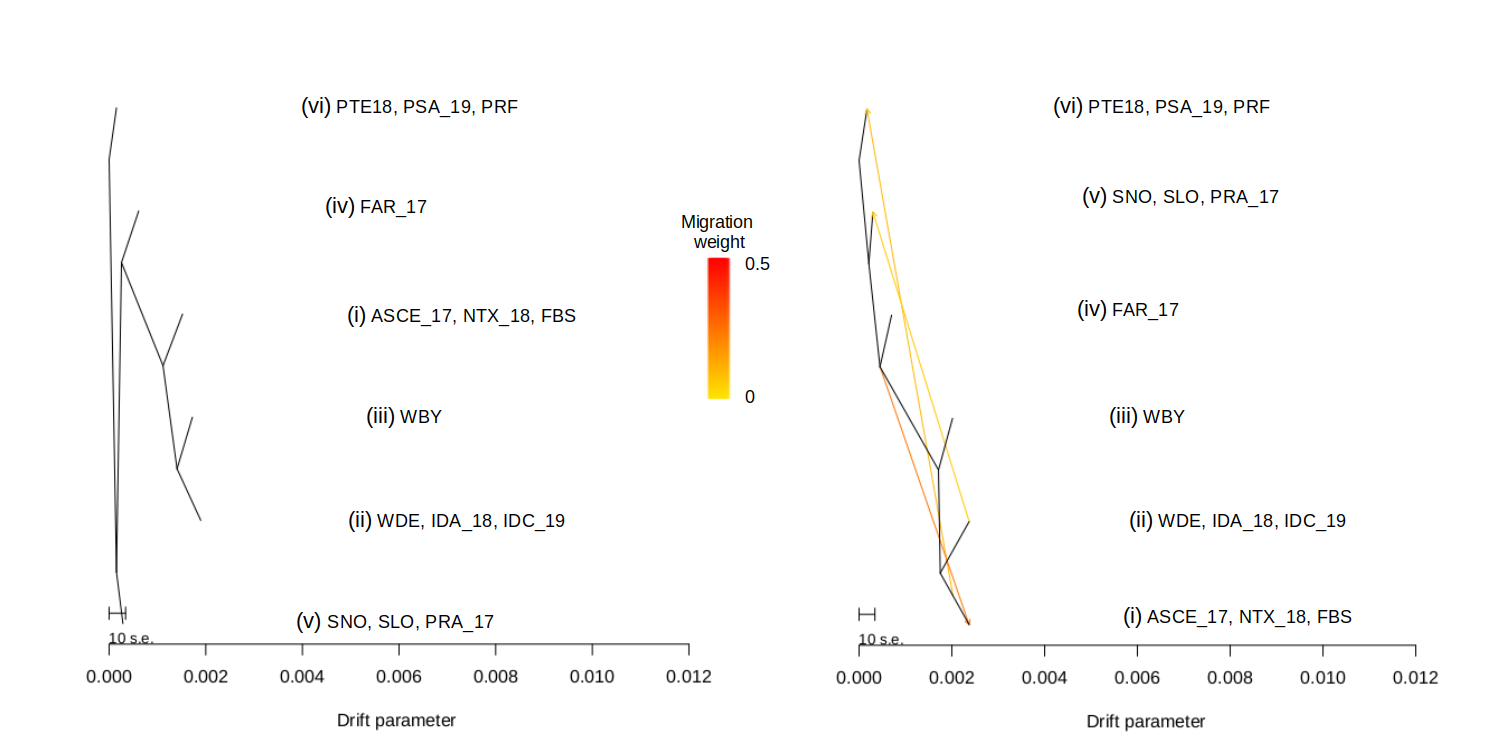


**Supplementary Figure 4.** Maximum-likelihood trees inferred by TREEMIX grouping the beds into the six main groups identified with fastSTRUCTURE using outlier dataset without (left) and with (right) migration events included. Migration events are depicted in heatmap coloured arrows. Bed codes are shown in Table 1.


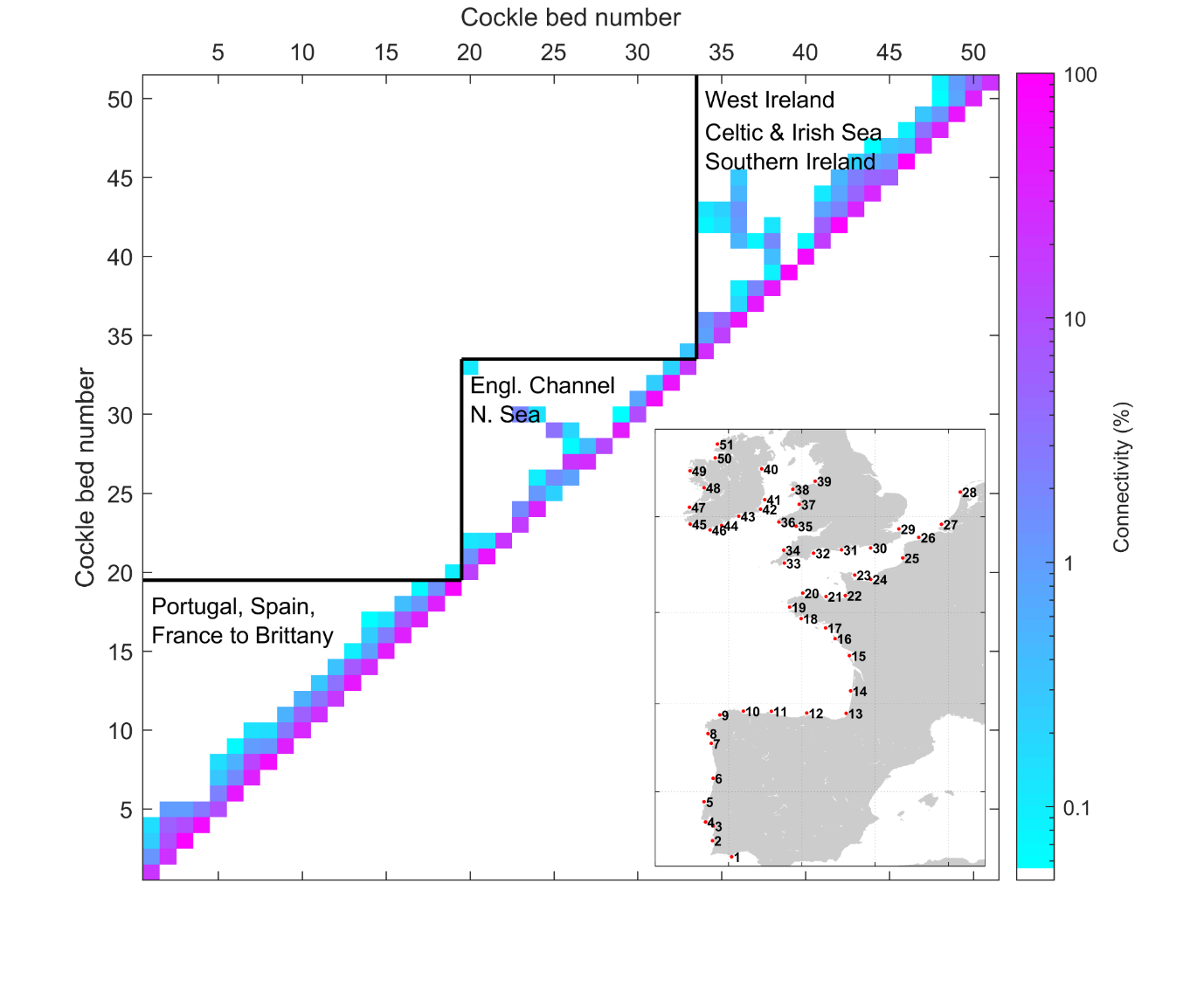


**Supplementary Figure 5.** Mean larval connectivity matrix for April to September from 2016 – 2018 for the mean of the 1 to 30 m depth releases. The strength of the connectivity between two sites is shaded and the location of each site can be seen in the map in the right-hand bottom corner. Distinct regions are indicated by the black lines.


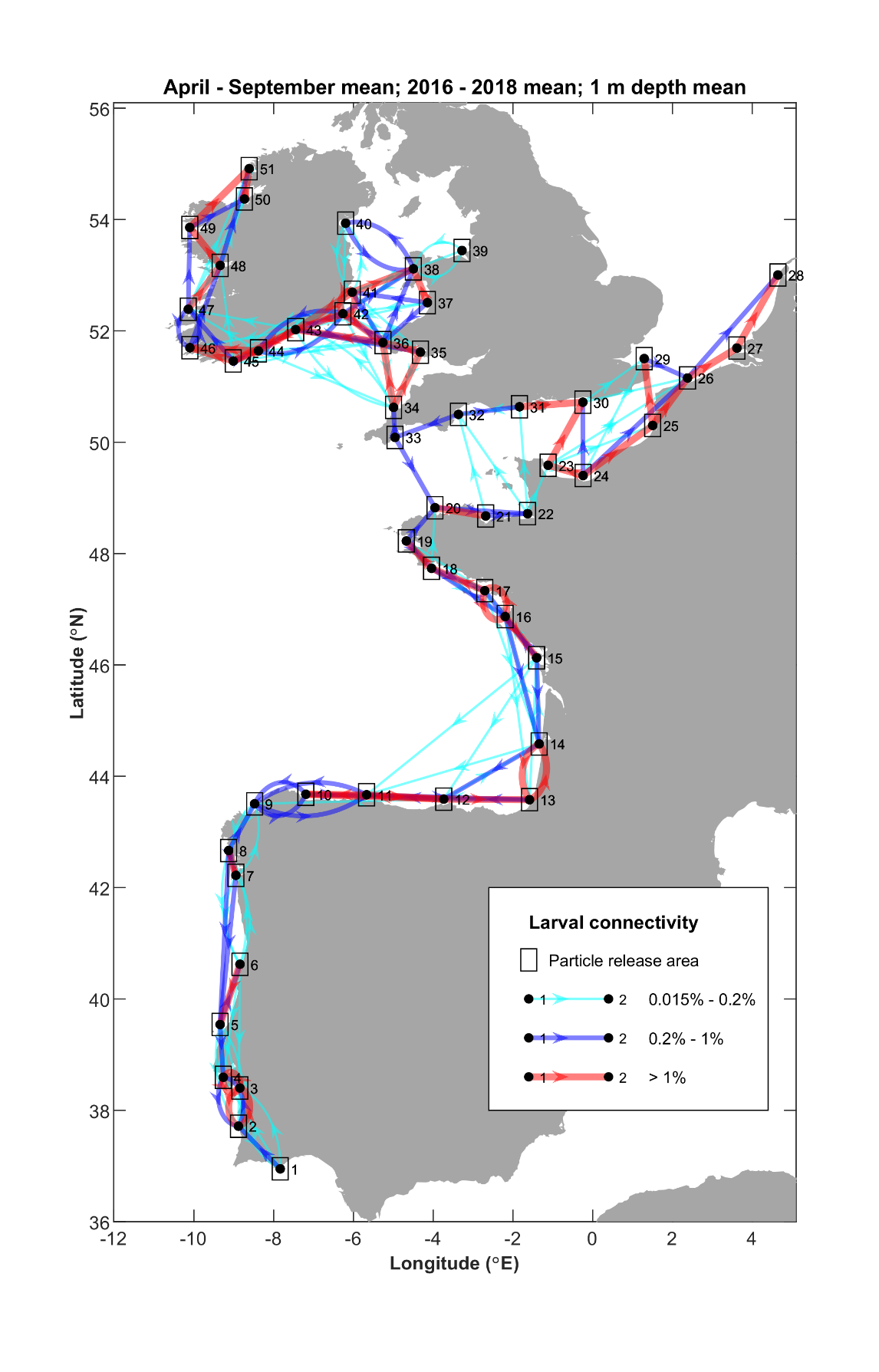


**Supplementary Figure 6.** Mean larval connectivity pathways for April to September from 2016 – 2018 releases 1 m depth. The direction of the arrows indicates the direction of larval transport and the colour and thickness of the connection displays the strength of the connection.


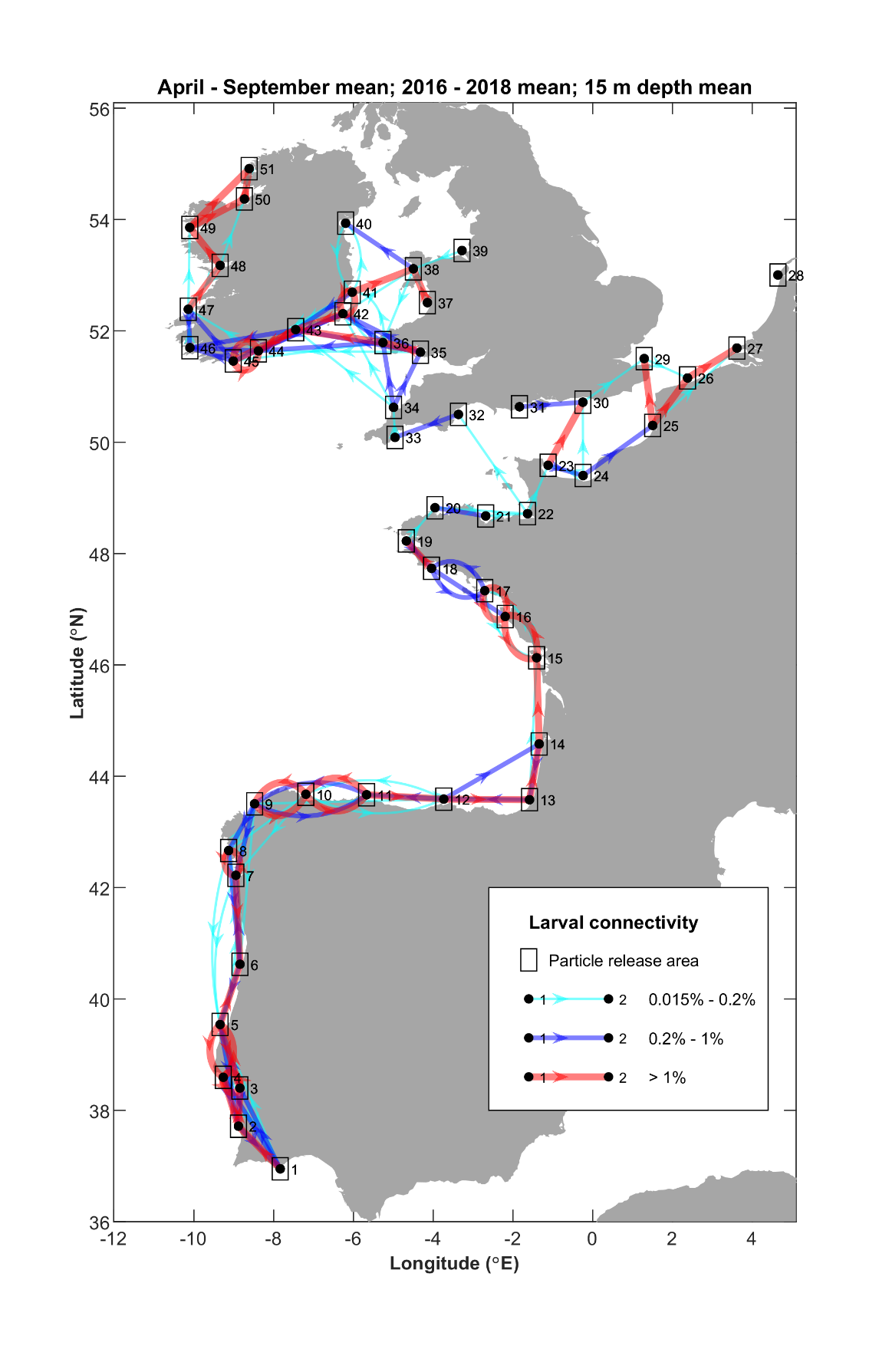


**Supplementary Figure 7.** Mean larval connectivity pathways for April to September from 2016 – 2018 releases 15 m depth. The direction of the arrows indicates the direction of larval transport and the colour and thickness of the connection displays the strength of the connection.


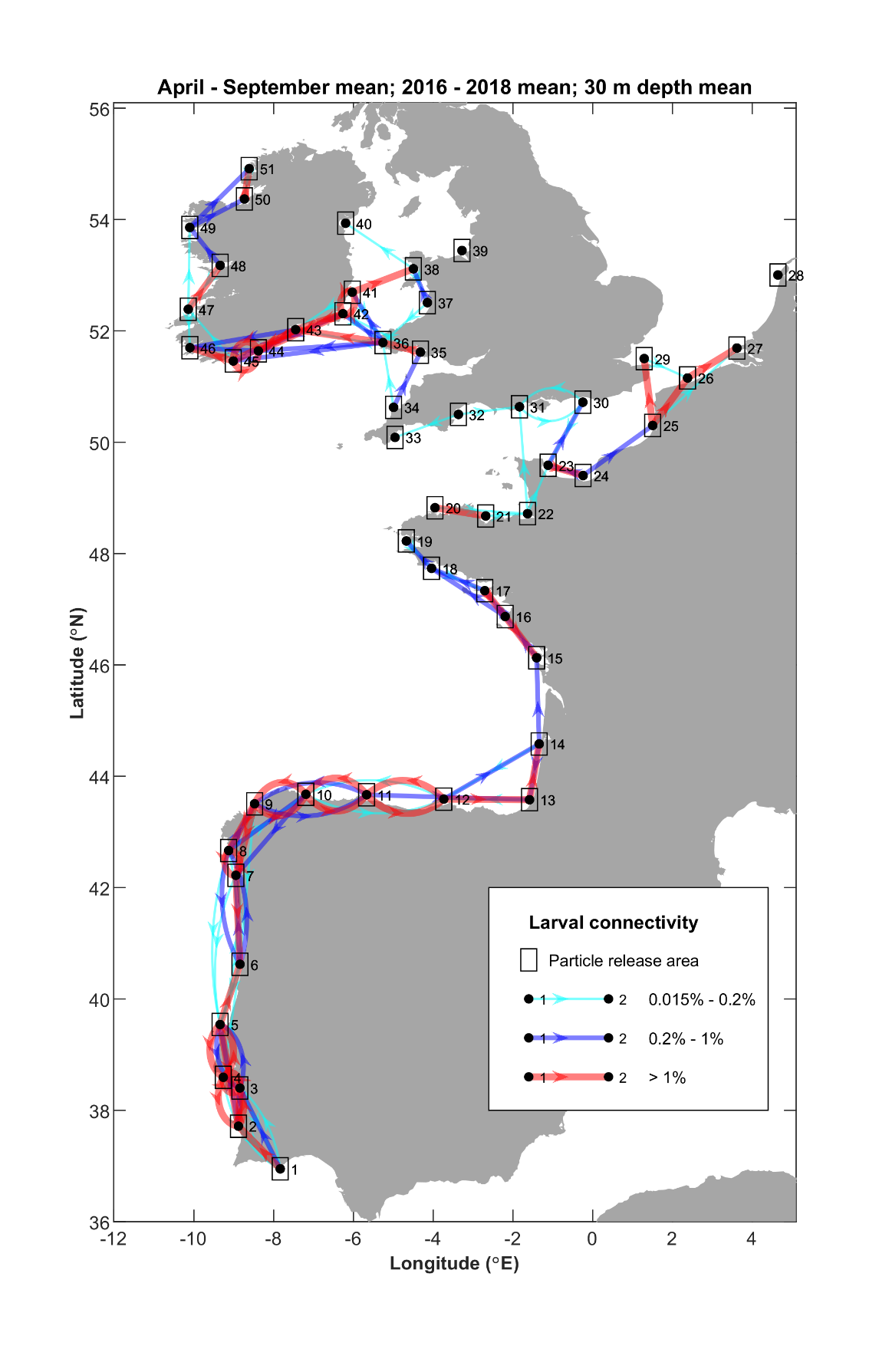


**Supplementary Figure 8.** Mean larval connectivity pathways for April to September from 2016 – 2018 releases 30 m depth. The direction of the arrows indicates the direction of larval transport and the colour and thickness of the connection displays the strength of the connection.


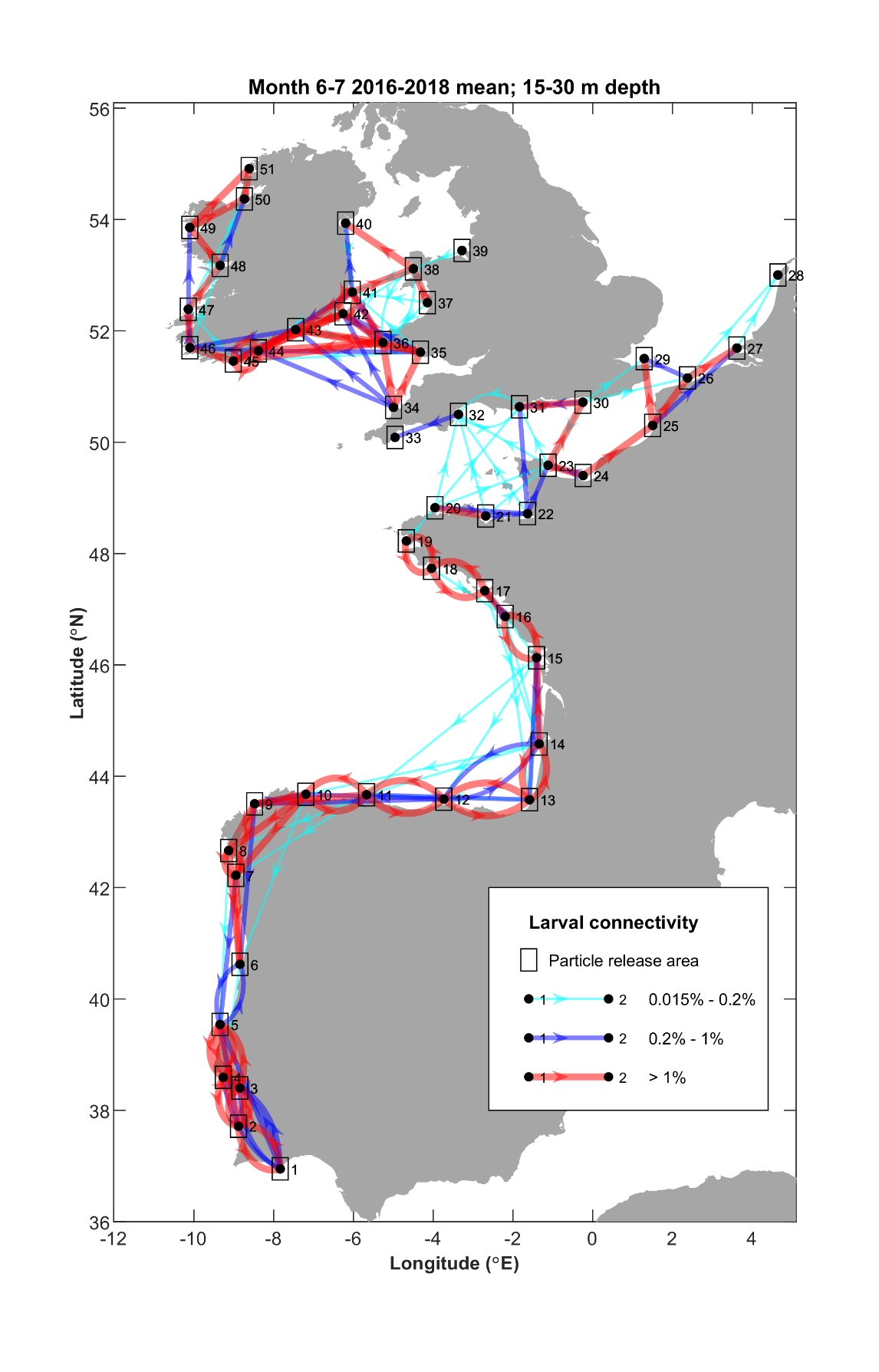


**Supplementary Figure 9.** Mean larval connectivity pathways for June to July from 2016 – 2018 releases for 15 and 30 m depth. The direction of the arrows indicates the direction of larval transport and the colour and thickness of the connection displays the strength of the connection.


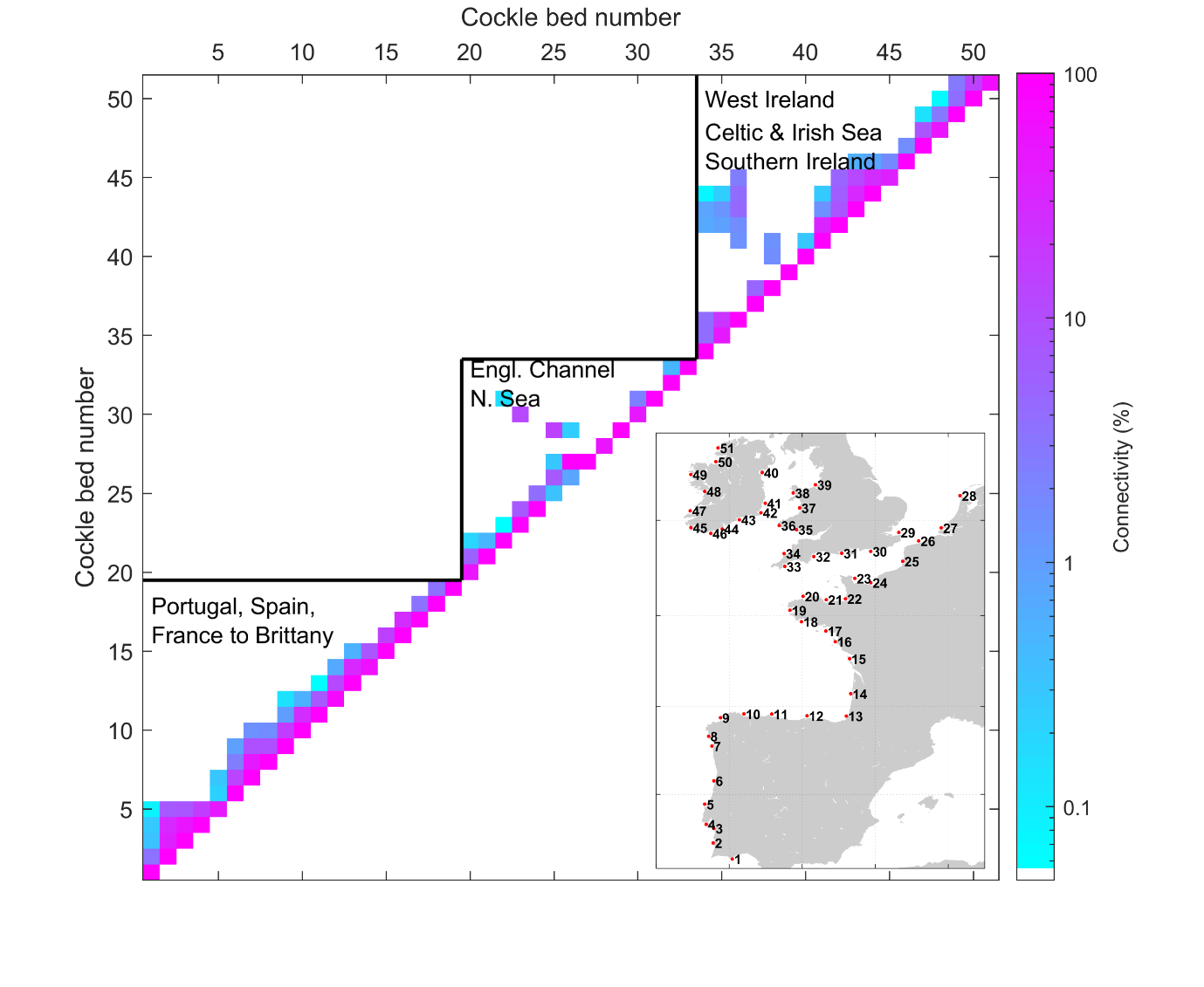


**Supplementary Figure 10.** Mean larval connectivity matrix for June to July from 2016 – 2018 for 15 and 30 m depth releases. The strength of the connectivity between two sites is shaded and the location of each site can be seen in the map in the right-hand bottom corner. Distinct regions are indicated by the black lines.
